# CO on a Rh/Fe_3_O_4_ single-atom catalyst: high-resolution infrared spectroscopy and near-ambient-pressure scanning tunnelling microscopy

**DOI:** 10.1039/d5fd00158g

**Published:** 2026-05-22

**Authors:** Nail El Hocine Barama, Chunlei Wang, Panukorn Sombut, David Rath, Adam Lagin, Martin Ormoš, Lena Puntscher, Faith J. Lewis, Zdeněk Jakub, Florian Kraushofer, Moritz Eder, Matthias Meier, Michael Schmid, Ulrike Diebold, Cesare Franchini, Peter Matvija, Jiří Pavelec, Gareth S. Parkinson

**Affiliations:** a Institute of Applied Physics, TU Wien 1040 Vienna Austria jiri.pavelec@tuwien.ac.at; b Department of Surface and Plasma Science, Charles University in Prague V Holesovickach 2 180 00 Praha Czech Republic; c Central European Institute of Technology (CEITEC), Brno University of Technology Brno 612 00 Czechia; d Faculty of Physics and Center for Computational Materials Science, University of Vienna Vienna AT 1090 Austria; e Dipartimento di Fisica e Astronomia, Università di Bologna Bologna IT 40126 Italy

## Abstract

Infrared reflection absorption spectroscopy (IRAS) offers a powerful route to bridging the materials and pressure gaps between surface science and powder catalysis. Using a newly developed IRAS setup optimised for dielectric single crystals, we investigate CO adsorption on the model single-atom catalyst Rh/Fe_3_O_4_(001). IRAS resolves three species: monocarbonyls at isolated, twofold-coordinated Rh adatoms, monocarbonyls at fivefold-coordinated Rh atoms embedded in the surface, and *gem*-dicarbonyls at isolated, twofold-coordinated Rh adatoms. Under ultra-high vacuum (UHV) conditions, RhCO monocarbonyl species at adatom sites dominate. Rh(CO)_2_*gem*-dicarbonyl formation is kinetically hindered and occurs predominantly through CO-induced dissociation of Rh dimers rather than sequential adsorption of two CO molecules at an isolated, twofold Rh adatom. The sequential-adsorption pathway to Rh(CO)_2_ becomes accessible at millibar CO pressures as evidenced by near-ambient-pressure scanning tunnelling microscopy (NAP-STM). These findings link the UHV behaviour to that expected under realistic reaction conditions. Assignments of the vibrational frequencies to individual species rely on isotopic labelling, thermal treatments, and a review of previous SPM, XPS, and TPD data, supported by density functional theory (DFT)-based calculations. While theory provides qualitative insight, such as the instability of dicarbonyls on fivefold-coordinated Rh atoms, it does not yet reproduce experimental frequencies quantitatively and is sensitive to the computational parameters, highlighting the need for robust experimental benchmarks. The spectroscopic fingerprints established here provide a reliable foundation for identifying Rh coordination environments in oxide-supported single-atom catalysts.

## Abstract 2

A model Rh/Fe_3_O_4_(001) single-atom catalyst enables site-resolved assignment of CO stretching frequencies.

## Graphical abstract

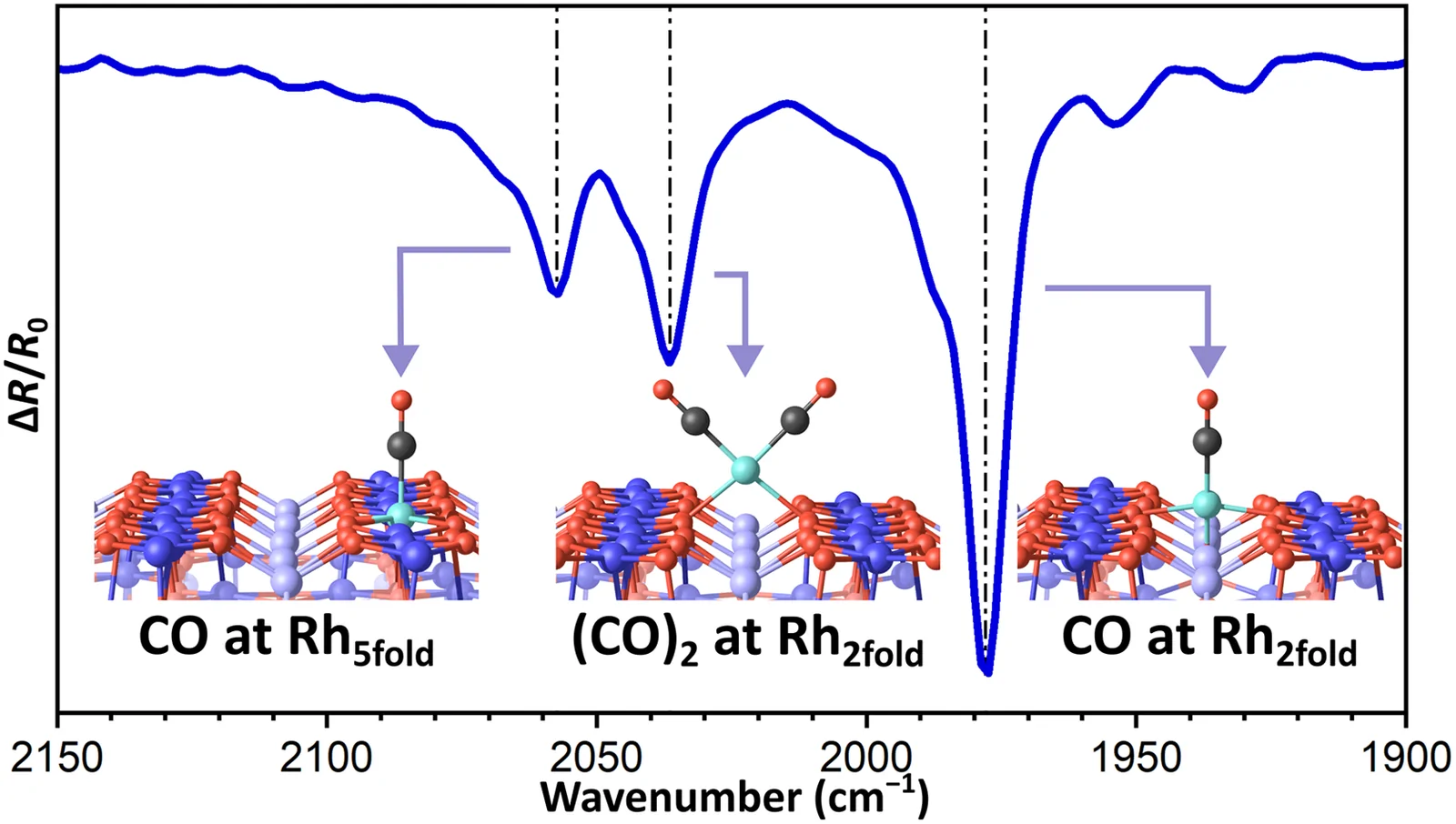

## 1. Introduction

Infrared (IR) spectroscopy is one of the most powerful techniques in heterogeneous catalysis and is the most widely used among optical spectroscopies in this field,^1^ as it can be used to monitor the evolution of surface-bound intermediates under *operando* conditions.^2–5^ Furthermore, different implementations of this technique enable its application to diverse types of samples, ranging from powders, investigated by diffuse reflectance infrared Fourier transform spectroscopy (DRIFTS), to flat single crystals, studied by infrared reflection absorption spectroscopy (IRAS or IRRAS). As a result, IR studies on powder-supported catalysts have provided invaluable insight into reaction mechanisms. Moreover, CO titration is frequently used to study the properties of active sites because the CO-stretch frequency is sensitive to the local coordination environment.^6–14^

Oxide-supported Rh compounds are key materials in heterogeneous catalysis, for example, in automotive emission control and various selective hydrogenation and oxidation reactions.^15–19^ Accordingly, IR absorption studies of CO adsorption on Rh-supported powder catalysts have attracted attention for decades. The earliest report dates back to 1957, when Yang and Garland investigated the sintering of dispersed Rh and assigned the resulting CO vibrational doublet to a single Rh adsorption site.^20^ Since then, numerous studies have employed IR spectroscopy to investigate Rh-based powder catalysts, revealing both the complexity of CO adsorption and the diversity of the resulting surface species.^18,21–28^ However, the structural complexity and heterogeneity of such catalysts make it difficult to unambiguously link a given vibrational feature to a specific atomic site. The coexistence of multiple adsorption geometries and the dynamic restructuring of active sites under reaction conditions further complicate interpretation, as highlighted in a recent review on IR characterisation of isolated atoms and nanoparticles.^29^

The surface-science approach enables experiments on well-defined catalytic model systems under tightly controlled UHV conditions. This includes single-atom catalysts (SACs) supported on single-crystal oxides, which provide a powerful platform for disentangling these complexities. Such model systems allow a direct correlation between atomic structure, electronic configuration, and vibrational signatures, whose frequencies are also, in principle, straightforward to calculate using DFT. Nonetheless, applying IRAS to metal oxide single crystals, the predominant support materials in catalysis, is challenging. The optimal incidence angles required to enhance adsorbate signals often coincide with regions of low infrared reflectivity, thereby drastically reducing signal intensity.^30^ As a result, IRAS studies on metal oxides remain scarce. A common workaround is to grow thin oxide films on metal substrates to benefit from the high reflectivity of metals;^31–34^ however, this strategy limits the accessible facets and may introduce structural or electronic deviations from those of bulk single crystals or conventional oxide powders. Overcoming these challenges for metal oxide single crystals is particularly important, as IRAS offers the unique combination of surface sensitivity and compatibility with elevated pressures, making it well suited to bridge the pressure gap between model catalysts and practical materials.^35,36^

While UHV-based surface-science studies provide insights at the atomic scale, they often cannot access adsorption pathways that are kinetically hindered at low pressure. In particular, adsorption steps that require short-lived intermediate geometries may be strongly suppressed under UHV conditions even when thermodynamically favourable. To assess how the CO adsorption mechanism evolves with pressure, and to test whether additional pathways become accessible under catalytic conditions, it is essential to complement UHV spectroscopy on well-investigated model systems with techniques capable of operating at elevated gas pressures. Adatoms of catalytic metals on the Fe_3_O_4_(001) surface represent a versatile model system. Fe_3_O_4_(001) exhibits a well-investigated subsurface cation-vacancy (SCV) reconstruction,^37^ which exposes a periodic array of surface oxygen atoms that can stabilise isolated metal adatoms. This makes Fe_3_O_4_(001) an ideal platform for SAC studies, and a wide range of metals, including Rh, have been systematically explored on this surface.^38–41^ In the case of Rh, previous surface-science work has established how preparation conditions such as adsorption temperature, annealing, and coverage determine whether Rh occurs as isolated adatoms, substitutional species, or small clusters.^39,42–45^ The interaction of CO with these Rh species has also been extensively characterised using experiments and DFT calculations, providing a detailed picture of the resulting adsorption geometries.^42,46,47^ As a result, Rh/Fe_3_O_4_(001) is one of the best-characterised model systems for oxide-supported Rh catalysts, offering atomic-level structural control that is difficult to achieve on powders. Investigating this system by IRAS is thus of particular interest, as it offers a direct link between fundamental surface-science insights and the behaviour of applied, high-surface-area catalysts.

In this work, we present a high-resolution IRAS investigation of CO adsorption on the model Rh/Fe_3_O_4_(001) single-atom catalyst. By combining isotope-substitution experiments (^12^CO, ^13^CO, and mixed ^12^CO/^13^CO) with precise control over Rh coverage and surface preparation, we assign each observed vibrational feature unambiguously to a specific Rh coordination environment. DFT calculations capture the qualitative trends, but do not reproduce the vibrational frequencies quantitatively. Our IRAS study, therefore, provides a set of reference spectra for oxide-supported single-atom catalysts, offering a firm foundation for interpreting CO vibrational signatures in practical Rh catalysts and for evaluating theoretical predictions. To probe the pressure dependence of the CO adsorption mechanism, we further complemented the UHV-IRAS measurements with NAP-STM, which allowed us to determine whether the sequential-adsorption pathway to Rh(CO)_2_, kinetically inaccessible in UHV, becomes accessible under mbar CO exposures.

## 2. Methods

IRAS measurements were performed using a newly developed setup designed to optimise the signal-to-noise ratio for metal oxide surfaces.^48^ All spectra were acquired with a resolution of 4 cm^−1^ and averaged over 4000 scans, corresponding to an acquisition time of approximately 20 min per spectrum. The measurements were conducted with p-polarised light at non-grazing incidence angles between 55° and 74°, a range optimised for the Fe_3_O_4_(001) surface to enhance peak intensity. The spectrometer used in the IRAS setup is a Bruker VERTEX 80v. The entire system is based on a custom-built UHV chamber designed for chemical reactivity studies, operating at a base pressure below 1 × 10^−10^ mbar.^49^ The chamber is equipped with a triple-pocket electron-beam evaporator for metal deposition and a temperature-stabilised quartz-crystal microbalance (QCM) used to calibrate the Rh deposition rate. For Fe_3_O_4_(001), one monolayer (1 ML) is defined as one Rh atom per (√2 × √2)R45° unit cell, corresponding to 1.42 × 10^14^ atoms per cm^2^. Carbon monoxide was dosed using a home-built molecular beam source that provides uniform exposure over a well-defined ≈3.5 mm diameter area on the sample surface.^49,50^ The UHV chamber is equipped with several complementary surface-science techniques, including X-ray photoelectron spectroscopy (XPS), ultraviolet photoelectron spectroscopy (UPS), temperature-programmed desorption (TPD), low-energy electron diffraction (LEED), and low-energy ion scattering (LEIS). In the present work, XPS was employed to verify surface cleanliness and confirm Rh coverages. XPS spectra were recorded using a monochromatized Al/Ag twin-anode X-ray source (SPECS XR 50 M, FOCUS 500) and a hemispherical analyser (SPECS Phoibos 150) with Al Kα radiation.

UHV scanning tunnelling microscopy (STM) measurements were performed on a separate Fe_3_O_4_(001) sample in an independent UHV system consisting of a preparation chamber (*p* < 1 × 10^−10^ mbar) and an analysis chamber (*p* < 7 × 10^−11^ mbar). The STM (Omicron µ-STM) was operated at room temperature in constant-current mode using electrochemically etched tungsten tips. Further details of this setup are provided in previous papers.^38,51,52^

Non-contact atomic force microscopy (nc-AFM) measurements were carried out on an additional Fe_3_O_4_(001) sample in a dedicated UHV chamber (*p* < 1 × 10^−10^ mbar) using a qPlus sensor^53^ (*f*_0_ = 31.8 kHz, *k* = 1800 N m^−1^, *Q* ≈ 10 000) with an electrochemically etched tungsten tip. The CO-tip functionalization was spontaneously formed during STM scanning or mild bias pulsing above Rh–CO species, similarly to previous reports.^40^

Near-ambient-pressure measurements were performed at the Nanomaterials Group at Charles University in Prague. The system consists of a UHV preparation chamber equipped with XPS, an electron-beam evaporator, an electron beam heating stage, and an oxygen supply, connected *via* a transfer line to a second chamber housing an Aarhus NAP-STM/AFM microscope. The base pressure was 1 × 10^−9^ mbar in the preparation chamber and 1 × 10^−10^ mbar in the STM/AFM chamber. CO (Linde, 99.997%) was introduced into the STM/AFM enclosure *via* a leak valve equipped with a cryogenic (LN_2_-cooled) filter. Near-ambient pressures in the range of 1–10 mbar were applied at room temperature and monitored using a calibrated Pirani gauge. STM images were acquired in constant-current mode under stable CO pressure, with typical tunnelling conditions of +0.7–1.2 V sample bias and 20–50 pA. After the NAP-STM measurements, the high-pressure cell was pumped back to UHV, and the sample was imaged again to confirm that no pressure- or tip-induced morphological changes had occurred.

All samples used in this study were natural Fe_3_O_4_(001) single crystals obtained from SurfaceNet GmbH. Surface preparation consisted of repeated cycles of Ne^+^ (or Ar^+^, depending on the chamber) ion sputtering at 300 K (1 keV, 15 min) followed by annealing under UHV at 900 K for 15 min. Every second annealing cycle was performed in an oxygen atmosphere (*p*(O_2_) = 3 × 10^−7^ mbar, 20 min) to prevent reducing the sample.

VASP was used for all DFT calculations with the PAW method and a 550 eV plane-wave cutoff.^54–56^ Electronic exchange–correlation was treated using the GGA-PBE functional with D3 dispersion corrections (Becke–Johnson damping), and strong correlation of Fe 3d states was described with *U*_eff_ = 3.61 eV.^57–60^ This choice of functional differs from the optB88-DF employed by Wang *et al.*,^46^ resulting in slightly different calculated adsorption energies. Electronic self-consistency and ionic-relaxation were converged to 10^−6^ eV and 0.02 eV Å^−1^, respectively. The Fe_3_O_4_(001) surface was modelled by an asymmetric 13-layer slab (7 octahedral and 6 tetrahedral Fe layers) in a (2√2 × 2√2)R45° supercell, with the top 4 layers relaxed, the bottom 9 fixed, Γ-point sampling only, and 14 Å of vacuum.^46^ CO vibrational frequencies were computed in the harmonic approximation using finite differences. Motivated by the use of DFT+U to mitigate self-interaction errors in localised d states, we also examined the effect of applying a Hubbard *U* to the Rh 4d states, using the same *U*_eff_ value as for the Fe 3d states, on the calculated CO stretching frequencies. The computed CO stretching vibrational frequency for adsorbed CO on the Rh/Fe_3_O_4_(001) was scaled by the factor *λ* = *v*^exp^_COgas_/*v*^cal^_COgas_ to compensate for the systematic DFT errors, where *v*^exp^_COgas_ and *v*^cal^_COgas_ are the experimental^61^ and calculated frequencies of an isolated CO molecule in the gas phase, respectively.

## 3. Summary of results from our previous studies

The adsorption of Rh on Fe_3_O_4_(001) in the low-coverage regime has been comprehensively characterised in our previous studies.^42,46,47^ Additional work by Sharp *et al.* further refined this picture by mapping the distribution of Rh species over a wide range of coverages and annealing temperatures.^39^ In the present study, we focus on low Rh coverages (<0.5 ML) deposited at room temperature, where isolated Rh adatoms dominate. These adatoms are twofold-coordinated (Rh_2fold_) to surface oxygen atoms within the SCV reconstruction and appear in STM images as bright protrusions centred between the rows of octahedrally coordinated Fe atoms (Fe_oct_).^42^ The adsorption energy of Rh_2fold_ was calculated to be *E*_ads_ = −4.42 eV.^46^ Occasionally, Rh_2fold_ adatoms pair to form Rh_2_ dimers, which appear as elongated protrusions in STM. At room temperature, the dimers oscillate between two equivalent configurations by moving parallel to the Fe_oct rows_.^42,46^ DFT calculations indicate that these dimers are thermodynamically preferred over two isolated adatoms by approximately 0.19 eV, but kinetic barriers prevent pairing of all Rh_2fold_ adatoms at room temperature. Dimers account for approximately 12% of the total Rh population at an initial coverage of 0.2 ML. In addition to adatoms and dimers, some Rh atoms substitute surface/subsurface Fe_oct rows_ sites within the spinel structure to form fivefold (Rh_5fold_) or sixfold (Rh_6fold_) coordinated Rh sites.^42^ These substitutional Rh atoms are stabilised relative to the Rh_2fold_ adatom by 0.67 eV for the fivefold configuration and by 1.10 eV for the sixfold subsurface configuration. Only the surface Rh_5fold_ species are relevant to our CO adsorption studies here. While these substitutional Rh atoms are already present in small quantities after room-temperature deposition, annealing above ≈400 K drives a substantial fraction of Rh_2fold_ adatoms into the more highly coordinated Rh_5fold_ sites.

**Fig. 1:**
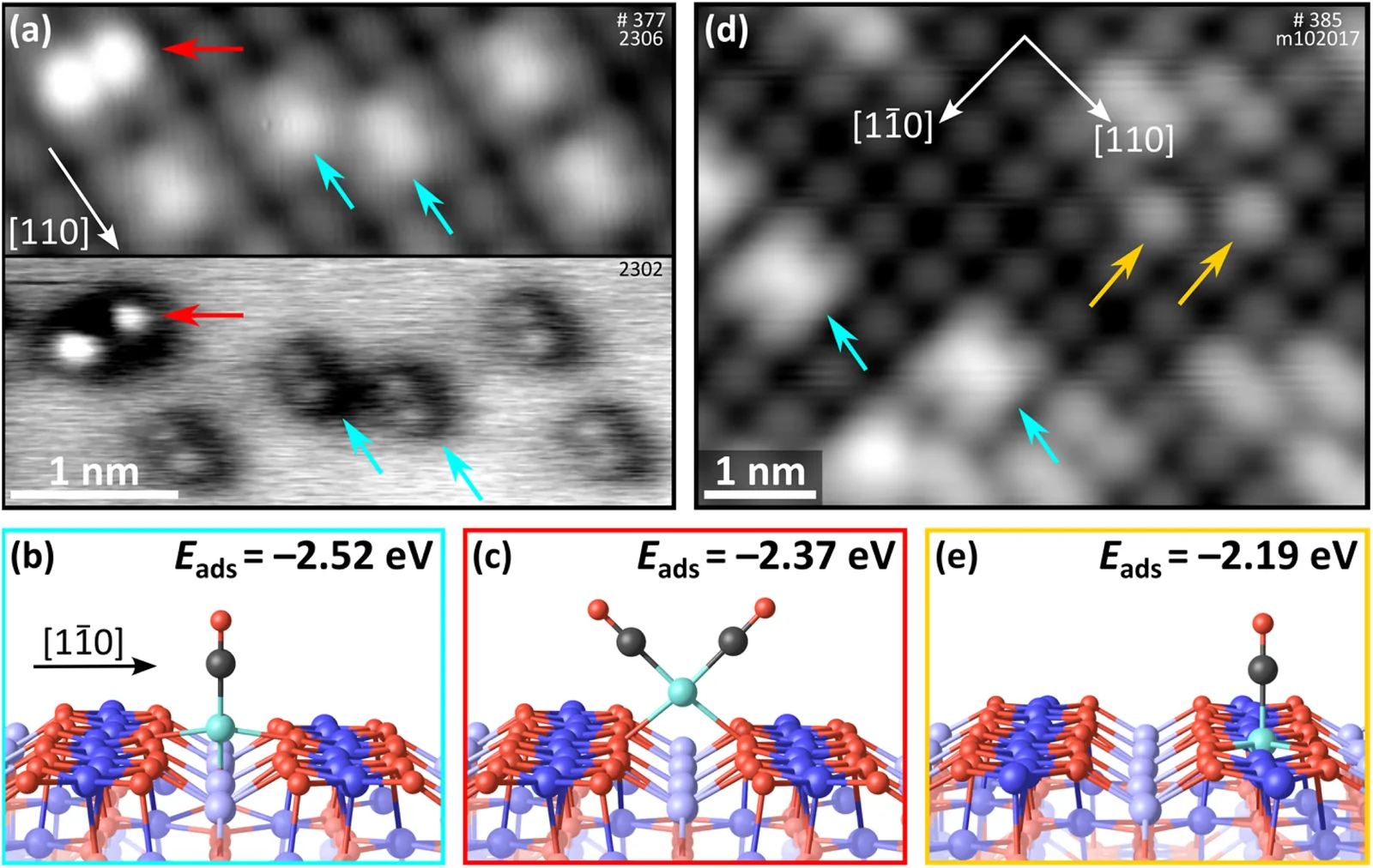
CO adsorption configurations on Rh/Fe_3_O_4_(001). (a) STM (top) and nc-AFM (bottom) images acquired at 78 K after CO exposure on low-coverage Rh/Fe_3_O_4_(001). Cyan arrows mark monocarbonyls on Rh_2fold_ sites, and red arrows indicate a *gem*-dicarbonyl on Rh_2fold_ sites. (b) DFT model of the pseudo-square-planar geometry adopted by the Rh_2fold_ monocarbonyl. The CO is not vertical but tilted towards the viewer by ≈20°. (c) DFT model of the *gem*-dicarbonyl on a Rh_2fold_ site.^46^ (d) STM image extracted from a movie recorded during CO exposure on 0.2 ML Rh/Fe_3_O_4_(001). Here, 1 monolayer (ML) corresponds to 1.42 × 10^14^ Rh atoms per cm^2^, *i.e.* one Rh atom per (√2 × √2)R45° surface unit cell of Fe_3_O_4_(001). Yellow arrows mark CO adsorbed on Rh_5fold_ atoms substituting Fe_oct_ sites. (e) DFT model of CO adsorbed on a Rh_5fold_ site. Adsorption energies are based on PBE-D3 with *U*_Rh_ = 0.

Fig. 1 summarises the three principal CO adsorption configurations on Rh sites observed after CO exposure on a low-coverage Rh/Fe_3_O_4_(001) surface.^42,46^Fig. 1a presents an STM image (top) and a nc-AFM image (bottom) of the same surface region, both recorded at 78 K using a CO-terminated tip after CO exposure. The most abundant species are Rh_2fold_CO monocarbonyls (highlighted by cyan arrows).^46^ Although CO adsorption modifies the local coordination of the Rh adatom, we retain the Rh_2fold_ notation throughout for simplicity, referring to the coordination of the Rh site prior to CO adsorption. Upon CO adsorption, the Rh_2fold_ adatom sinks slightly into the surface and forms an additional bond with one of the two equivalent subsurface oxygen atoms, forming a pseudo-square-planar geometry tilted away from the surface normal. The weak bond to the subsurface oxygen enables rapid switching between two equivalent configurations along the [110] direction. The calculated switching barrier is approximately 0.1 eV.^46^ As a result, at room temperature as well as at 78 K, the species appear as faint double protrusions in nc-AFM and as elongated features in STM. The corresponding structural model and calculated adsorption energy are shown in Fig. 1b.

In addition, the bright double-lobed protrusions oriented perpendicular to the Fe_oct_ rows (red arrows in Fig. 1a) are assigned to Rh_2fold_(CO)_2_*gem*-dicarbonyl species. As demonstrated by Wang *et al.*,^46^ these dicarbonyls form almost exclusively through CO-induced dissociation of the minority Rh_2_ dimers present on the surface under UHV conditions. This process was captured in sequential STM images, and DFT calculations revealed that, upon CO exposure, the Rh_2_ dimers pass through an unstable intermediate Rh_2_(CO)_3_ configuration, which subsequently dissociates to produce one monocarbonyl Rh_2fold_CO and one *gem*-dicarbonyl Rh_2fold_(CO)_2_. Although the Rh_2fold_(CO)_2_ configuration is thermodynamically stable (average adsorption energy *E*_ads_ = −2.37 eV per CO with the PBE-D3 functional used in the current work; no *U* on Rh), sequential adsorption of two CO molecules on an isolated Rh_2fold_ adatom was never observed in UHV. Our previous combined STM/DFT analysis suggested that this pathway requires the Rh_2fold_CO to adopt a transient, sterically constrained geometry with an extremely low configurational probability. Since the Rh_2fold_CO assumes this configuration only during a tiny fraction of the time (<10^−8^), adsorption of another CO during these short periods is extremely unlikely at UHV gas impingement rates.^46^ This kinetic picture predicts that sufficiently high CO pressures should enable the sequential-adsorption mechanism by dramatically increasing the rate at which CO molecules encounter Rh_2fold_CO species in the required transient geometry. As shown in section 5, NAP-STM measurements presented in the current work indeed reveal that the sequential CO adsorption pathway becomes accessible under mbar CO pressures, providing direct experimental support for this kinetic interpretation. The structural model of Rh_2fold_(CO)_2_ and the calculated adsorption energy per CO molecule are shown in Fig. 1c.

CO can also adsorb on the substitutional Rh_5fold_ sites. In this case, DFT calculations indicate that *gem*-dicarbonyl formation is energetically unfavourable.^46^ Only a single CO molecule can bind to a Rh_5fold_ atom, completing an octahedral coordination environment (Fig. 1e). CO adsorption at these sites is significantly weaker (*E*_ads_ = −2.19 eV with PBE-D3, *U*_Rh_ = 0) than at the Rh_2fold_ sites (*E*_ads_ = −2.52 eV).^46^ These calculations are in excellent agreement with CO TPD experiments, where monocarbonyls on Rh_5fold_ lead to a desorption peak at around 410 K, notably lower than the desorption peak stemming from the Rh_2fold_ at 530 K.^42^ The STM image in Fig. 1d is extracted from a movie recorded before and during CO exposure (included as Movie S1). The movie illustrates how Rh_2fold_ sites transform into elongated features upon CO adsorption (highlighted again by cyan arrows).^46^ In contrast, the Rh_5fold_ sites exhibit only a subtle change in contrast upon CO adsorption, and the CO does not appear elongated (no flipping between two configurations; yellow arrows in Fig. 1d). Owing to their low concentration under the present deposition conditions (temperature and coverage), these species are observed only rarely in STM. Nevertheless, they are expected to give distinct signatures in the infrared spectra, as IRAS is very sensitive to dipole moments normal to the surface. In principle, low-temperature infrared spectroscopy could also be used to probe dimer carbonyl intermediates, provided the dimers are stabilized under the chosen conditions. In the present work, we focus on the three adsorption configurations observed at room temperature. Together, these three configurations—monocarbonyls on Rh_2fold_, *gem*-dicarbonyls on Rh_2fold_, and monocarbonyls on Rh_5fold_—constitute the structural basis for interpreting the IRAS spectra discussed in Section 4. Their distinct adsorption geometries and adsorption energies are expected to produce characteristic shifts in the CO stretching frequencies that allow each configuration to be distinguished spectroscopically, and they represent the three relevant Rh–CO states from which the kinetic behaviour under different CO pressures emerges. Section 5 provides a direct NAP-STM test of this kinetic picture.

## 4. IRAS of CO on Rh/Fe_3_O_4_(001)

### 4.1. Three different CO species

**Fig. 2:**
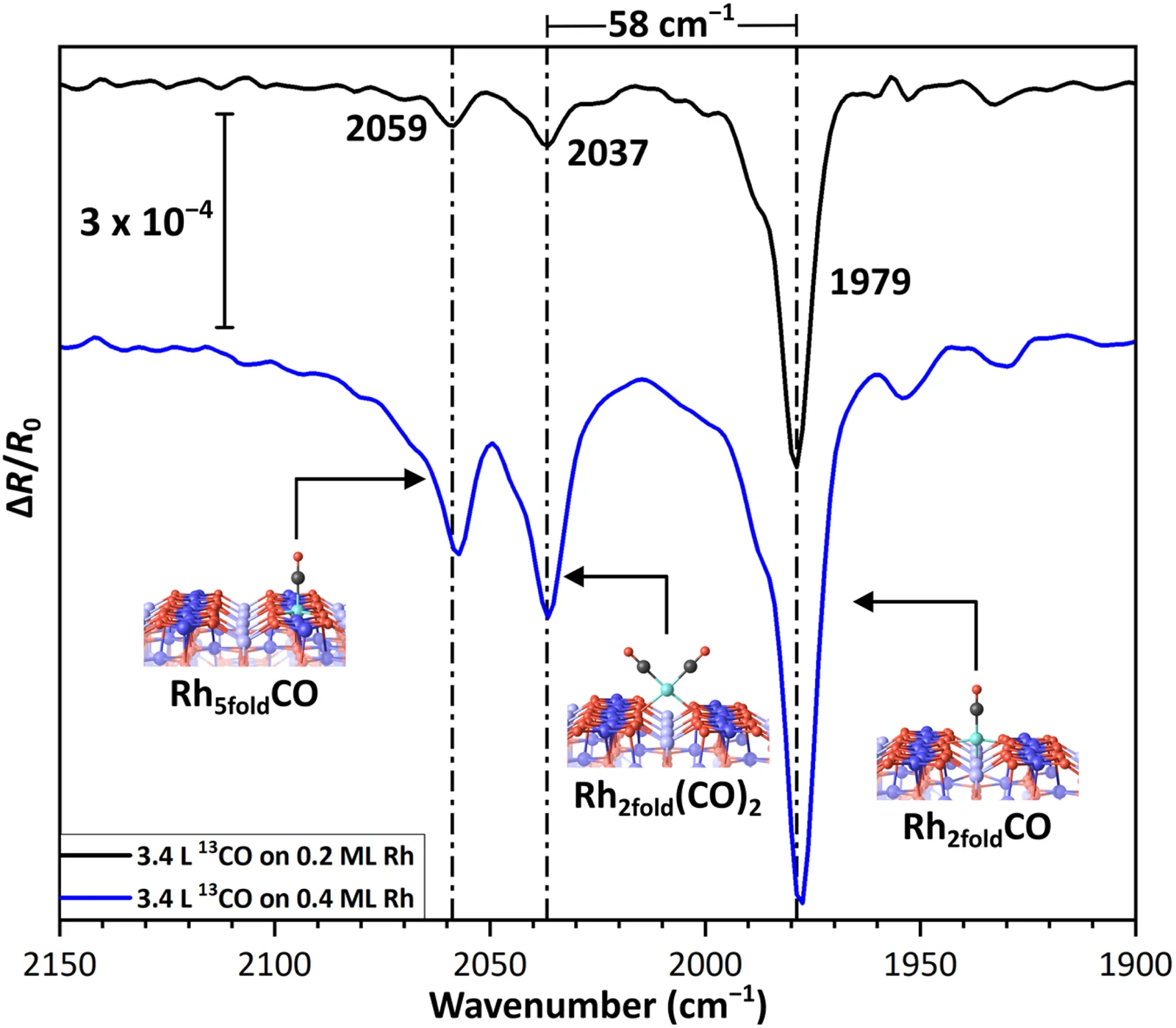
High-resolution IRAS spectra of ^13^CO adsorbed on Rh/Fe_3_O_4_(001). p-polarised IRAS spectra of Rh-decorated Fe_3_O_4_(001) with Rh coverages of 0.2 ML (black) and 0.4 ML (blue) after 3.4 L ^13^CO exposure. ^13^CO was introduced to the surface at room temperature, and the IRAS spectra were collected under identical conditions at *T* = 300 K. Three distinct vibrational bands are observed at 1979, 2037, and 2059 cm^−1^; their intensities increase with Rh coverage. These features are assigned to CO adsorbed on twofold-coordinated Rh_2fold_ sites, the symmetric stretch of *gem*-dicarbonyl Rh_2fold_(CO)_2_ species, and CO adsorbed on Rh_5fold_ sites, respectively. Spectra were acquired with 4 cm^−1^ resolution and averaged over 4000 scans (≈20 min acquisition time per spectrum).

To enable a direct comparison with the STM and DFT results summarised in Fig. 1, IRAS measurements were performed on a Rh-decorated Fe_3_O_4_(001) sample with Rh coverages that had been previously characterised in detail.^42,46^Fig. 2 shows p-polarised IRAS spectra acquired after exposure to 3.4 langmuirs (L; 1 L = 1.33 × 10^−6^ mbar s) of ^13^CO at room temperature for two different Rh coverages, 0.2 ML (black spectrum) and 0.4 ML (blue spectrum). In all experiments, the reference spectrum *R*_0_ was recorded after Rh deposition but prior to CO dosing at 300 K, ensuring that only CO-induced changes appear in the spectra. For 0.2 ML Rh, three distinct absorption bands are observed in the CO-stretching region at 1979, 2037, and 2059 cm^−1^. All three features are also present at 0.4 ML Rh and increase in intensity with increasing Rh coverage, indicating that each originates from CO adsorbed on Rh-related sites. The positions of the three bands remain nearly unchanged in the 0.4 ML spectrum, which suggests that dipole–dipole interactions between neighbouring CO molecules are negligible. This is consistent with the previous STM studies,^42,46^ which show that the nearest-neighbour distance between Rh_2fold_ adatoms on Fe_3_O_4_(001) is fixed by the surface reconstruction at 8.4 Å.

Based on the CO stretch frequencies calculated by DFT (Table 1), we assign the low-frequency peak at 1979 cm^−1^ to CO adsorbed on the Rh_2fold_ sites (Fig. 1b). The relatively high intensity of this band is consistent with STM results, which show that Rh_2fold_CO species are by far the most abundant configuration containing CO under these conditions.^46^ We note, however, that IRAS peak intensities are not solely determined by the population of a given species, but also depend on the magnitude and orientation of the dynamic dipole moment relative to the surface normal, and on the detailed bonding geometry.

**Table 1:** Experimental and calculated C–O stretching frequencies, together with calculated CO adsorption energies for the different adsorption configurations. The calculated values are obtained by DFT with and without Hubbard *U* correction applied to the Rh 4d states

Configuration	*E*_ads_, CO (eV)	*ν̃*_DFT_,^13^CO (cm^−1^)	*ν̃*_exp_,^13^CO (cm^−1^)	*ν̃*_DFT_,^12^CO (cm^−1^)	*ν̃*_exp_,^12^CO (cm^−1^)
***U***_**eff, Rh**_ **= 0**					
Rh_2fold_CO	−2.52	1971	1979	2021	2026
Rh_2fold_(CO)_2_	−2.37	2022 (*sym*)	2037	2076 (*sym*)	2085
		1961 (*asym*)		2012 (*asym*)	
Rh_5fold_CO	−2.19	2025	2059	2078	2106

***U***_**eff, Rh**_ **= 3.61 eV**					
Rh_2fold_CO	−1.93	1978	1979	2029	2026
Rh_2fold_(CO)_2_	−2.03	2023 (*sym*)	2037	2076 (*sym*)	2085
		1974 (*asym*)		2025 (*asym*)	
Rh_5fold_CO	−1.77	2047	2059	2100	2106

The two higher-frequency bands at 2037 cm^−1^ and 2059 cm^−1^ appear with much lower intensity than the dominant 1979 cm^−1^ band in both spectra. As mentioned previously, only small populations of Rh_2_ dimers (the precursors of *gem*-dicarbonyls) and Rh_5fold_ sites are present at this coverage. This low abundance is reflected in the correspondingly weak intensity of these two features. Based on DFT, the assignment of these two peaks is uncertain since the calculation without *U* on the Rh 4d states yields almost identical frequencies for the symmetric mode of the *gem*-dicarbonyl and the CO at the Rh_5fold_ embedded in the surface. In the following, we will demonstrate that the assignment of the experimental IR bands in Fig. 2 and Table 1 is correct, although the agreement between DFT and experiment is worse than for the Rh_2fold_CO.

In principle, one could also consider whether one of the two high-frequency IRAS peaks originates from CO adsorbed on small Rh clusters. However, at a coverage <0.5 ML Rh, the amount of Rh clusters is negligible.^39^ Moreover, because clusters exhibit a wide distribution of sizes and geometries, their vibrational signatures are typically broad rather than sharp, unlike the well-defined peaks observed in Fig. 2. Finally, CO adsorbed on small Rh clusters normally exhibits lower stretching frequencies (≈2040 cm^−1^ for ^12^CO).^62^

Table 1 shows that the CO stretch frequencies are directly correlated to the adsorption energies. A strong Rh–CO interaction is associated with back-donation from Rh 4d into the CO 2π* orbital, weakening the C–O bond and shifting its stretching frequency to lower values.^63–66^

### 4.2. Annealed Rh/Fe_3_O_4_(001): increase of Rh_5fold_ sites

**Fig. 3:**
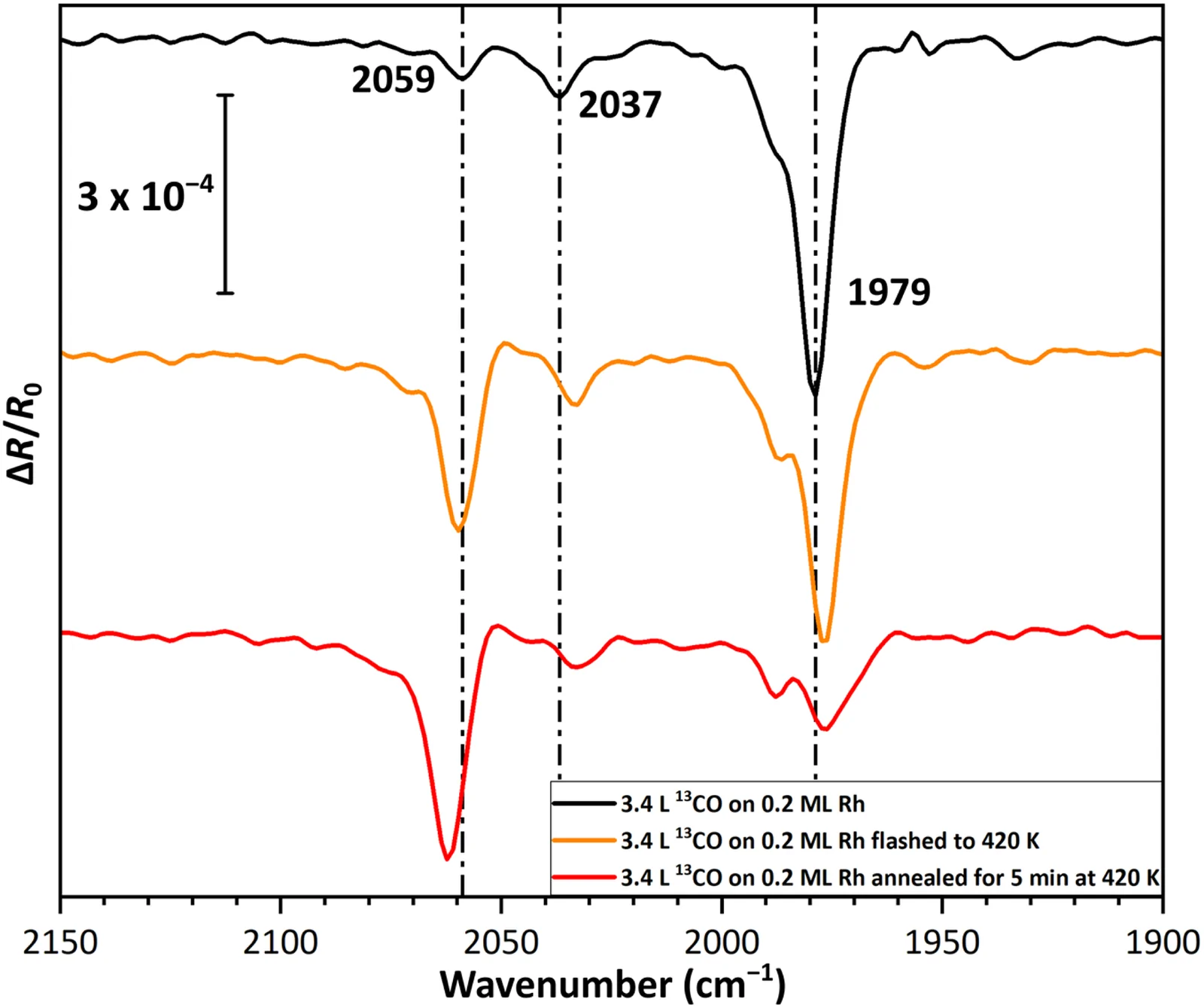
Thermally induced conversion of twofold to fivefold Rh sites monitored by IRAS: p-polarised IRAS spectra of Rh-decorated Fe_3_O_4_(001) surfaces (initial Rh coverage 0.2 ML) subjected to different thermal treatments prior to 3.4 L ^13^CO exposure. The black spectrum corresponds to the as-deposited sample kept at 300 K (same as Fig. 2). The orange spectrum was obtained after a brief flash to 420 K followed by cooling to 300 K, while the red spectrum was recorded after annealing the surface for 5 min at 420 K. All spectra were measured at 300 K after CO exposure.

As shown in previous STM and XPS studies,^39,42^ Rh_2fold_ adatoms are stable only below ≈400 K. Upon heating above this temperature, these species begin to incorporate into the surface lattice, forming fivefold and sixfold Rh sites. This structural transformation should be detectable by IRAS, as CO molecules adsorbed on twofold and fivefold Rh sites exhibit distinct C–O stretching frequencies, whereas Rh_6fold_ is below the surface and therefore not capable of CO adsorption. Fig. 3 presents p-polarised IRAS spectra of Rh/Fe_3_O_4_(001) surfaces that all had the same initial Rh coverage of 0.2 ML but underwent different thermal treatments prior to 3.4 L ^13^CO dosing, leading to a different distribution of Rh species. The black spectrum corresponds to the as-deposited sample, which was kept at room temperature, and is identical to the one shown in Fig. 2.

For the orange spectrum, the sample was briefly flashed to ≈420 K after Rh deposition and then cooled back to 300 K. The spectrum shows a pronounced increase in the intensity of the high-frequency band at 2059 cm^−1^, consistent with its assignment to monocarbonyls on Rh_5fold_ sites that form upon thermal conversion of Rh_2fold_ species. The accompanying decrease in the low-frequency peak further supports this interpretation. The shoulder of the peak at 1979 cm^−1^ is attributed to CO adsorbed on Rh_2fold_ adatoms located at defect sites, primarily antiphase domain boundaries (APDBs) and sites above additional subsurface Fe_oct_ cations, as described in our previous studies.^67,68^ Such defect sites can also stabilise Rh adatoms in a twofold-coordination with a slightly higher adsorption energy, resulting in a weaker Rh–CO interaction and consequently a blue shift of the C–O stretching frequency. The shoulder appears more distinct in the orange spectrum mainly because the 1979 cm^−1^ peak undergoes a slight red shift after flashing to ≈420 K. A similar small shift is observed for the intermediate band at 2037 cm^−1^. These small shifts likely reflect subtle changes in the Rh–CO interaction strength due to modest modifications in the electronic structure of the surface and subsurface layers. Such changes can accompany structural rearrangements in which some Rh_2fold_ adatoms convert into fivefold surface species, while others incorporate into subsurface positions to form Rh_6fold_, as seen previously for Rh and other transition metal adatoms.^38,42^ The nearly unchanged intensity of the shoulder compared to the black spectrum indicates that regular twofold Rh_2fold_ species are the first to convert into Rh_5fold_, while those associated with defects remain stable after this brief heat treatment.

The red spectrum in Fig. 3 was recorded at 300 K after annealing the surface for 5 minutes at 420 K. This longer temperature treatment further decreases the intensity of both the low-frequency Rh_2fold_CO band and its shoulder, while the high-frequency band assigned to CO on Rh_5fold_ becomes more intense. This provides direct experimental support for the peak assignments, as it is consistent with earlier STM and XPS observations of the evolution of Rh species under comparable annealing conditions.^39,42^ In contrast to the behaviour of the twofold band, the fivefold band shows a slight blue shift after annealing, likely reflecting a modest reduction in π-backdonation from Rh_5fold_ to CO as they become more tightly integrated into the surface lattice. The resulting weaker Rh–CO bond leads to a slightly higher C–O stretching frequency, in agreement with the observed shift.

Although the shift in intensity from the low- to the high-frequency band provides clear qualitative evidence for their assignment to CO on twofold and fivefold Rh sites, respectively, it remains difficult to determine quantitatively how many Rh_2fold_ atoms convert into Rh_5fold_ upon annealing. This is because CO binds in different geometries on the two sites, leading to different orientations of the vibrational dipole moment relative to the surface, and, possibly, different magnitudes of the dipole moment. CO adsorbed on Rh_5fold_ stands upright, so its dipole moment change is normal to the surface and couples strongly to the perpendicular component of p-polarised light. In contrast, CO bound to Rh_2fold_ adopts a pseudo-square-planar geometry and is tilted, resulting in both perpendicular and in-plane components of the dipole moment. Our measurements were conducted with p-polarised light at non-grazing incidence angles below 74°. At these incidence angles, perpendicular dipoles produce strong negative features, whereas in-plane dipoles give much weaker positive signals (approximately one-fifteenth of the perpendicular intensity if the dipole is parallel to the incidence plane, and no signal in p polarisation for the case of a dipole moment normal to the incidence plane; see Fig. S1). Consequently, the intensities of the two peaks are not a quantitative measure of the abundance of these species.

### 4.3. Isotopic confirmation of the *gem*-dicarbonyl band

**Fig. 4:**
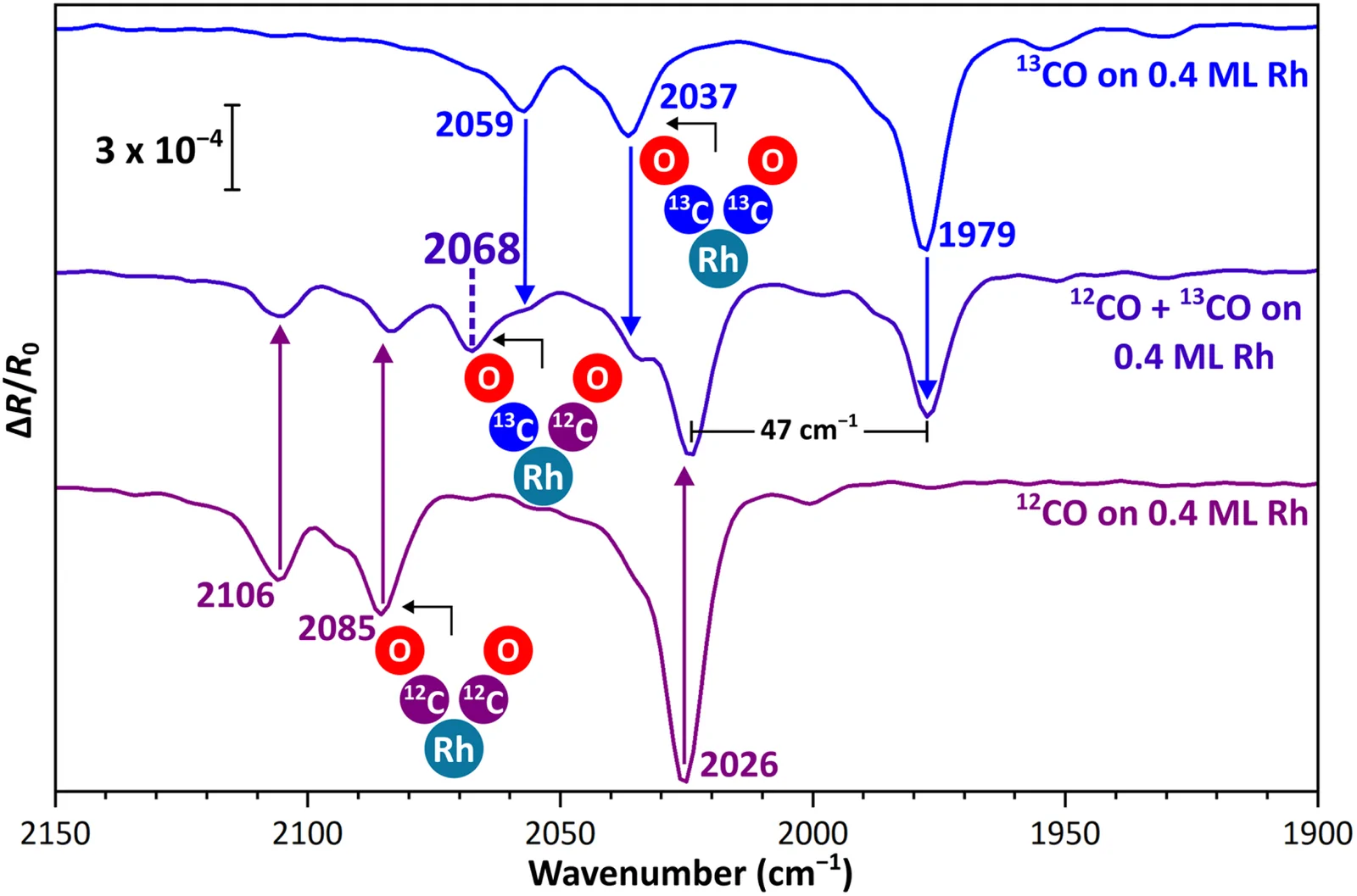
Isotopic confirmation of the *gem*-dicarbonyl assignment. p-polarized IRAS spectra recorded at 300 K after exposing 0.4 ML Rh/Fe_3_O_4_(001) to ^13^CO (blue), ^12^CO (purple), and an equimolar ^12^CO/^13^CO mixture (dark violet). Each pure-isotope spectrum exhibits three bands corresponding to CO adsorbed on twofold and fivefold Rh sites, as well as the symmetric stretch of the Rh_2fold_(CO)_2_*gem*-dicarbonyl. In the ^12^CO spectrum, all ^13^CO features are shifted to lower frequency by ≈47 cm^−1^, consistent with the expected isotopic dependence of the C–O stretching mode. In the mixed-isotope experiment, an additional intermediate band appears between the pure-^12^CO and pure-^13^CO *gem*-dicarbonyl peaks, arising from vibrational coupling within mixed-isotope Rh_2fold_(^13^CO)(^12^CO) species at 2068 cm^−1^, see sketch. The presence of this intermediate feature provides unambiguous evidence that the mid-frequency band at ≈2037 cm^−1^ (for ^13^CO) originates from a *gem*-dicarbonyl species rather than a monocarbonyl. The relative intensities of the three *gem*-dicarbonyl peaks in the mixed-isotope spectrum are consistent with the expected 1 : 2 : 1 statistical distribution for an equimolar ^12^CO/^13^CO mixture.

To provide direct experimental confirmation of the assignment of the mid-frequency peak to *gem*-dicarbonyl Rh_2fold_(CO)_2_ species, isotopic substitution experiments were performed using ^12^CO, ^13^CO, and an equimolar ^12^CO/^13^CO mixture on identically prepared 0.4 ML Rh/Fe_3_O_4_(001) surfaces. Fig. 4 presents the corresponding p-polarised IRAS spectra. The top (blue) spectrum corresponds to ^13^CO, identical to that shown in Fig. 2; the bottom (purple) to ^12^CO; and the middle (dark violet) to the ^12^CO/^13^CO mixture.

As expected, the pure ^12^CO and ^13^CO spectra each display three absorption bands of comparable relative intensity, with all ^13^CO features shifted to lower frequencies by ≈47 cm^−1^ relative to the other isotope. This isotopic shift is consistent with the change in the reduced mass of the C–O system and is in excellent agreement with the harmonic-oscillator model. For monocarbonyl species adsorbed on Rh atoms, exposure to an equimolar mixture of ^12^CO and ^13^CO should yield two peaks of similar intensity separated by ≈47 cm^−1^, corresponding to identical Rh sites bonded to either isotope. This behaviour is indeed observed in the mixed-isotope (dark violet) spectrum for the two peaks at 1979 and 2059 cm^−1^ with ^13^CO (2106 and 2026 cm^−1^ with ^12^CO), confirming their assignment as monocarbonyl Rh–CO species. In contrast, *gem*-dicarbonyls behave differently: co-adsorption of ^12^CO and ^13^CO should produce a third, intermediate band between the pure-isotope peaks, arising from mixed-isotope Rh_2fold_(^12^CO)(^13^CO) species. The appearance of such an intermediate band is a well-established fingerprint of intramolecular vibrational coupling between the two CO ligands, as reported previously by Frank *et al.*^26^

In the present case, the mixed-isotope spectrum reveals a new feature at 2068 cm^−1^, providing solid evidence that the mid-frequency band in the pure-isotope spectra originates from Rh_2fold_(CO)_2_ rather than from distinct monocarbonyl species. The measured peak separations between the symmetric ^12^CO and mixed-isotope bands (≈17 cm^−1^), and between the mixed-isotope and ^13^CO bands (≈31 cm^−1^), are fully consistent with the previously reported coupling strengths for *gem*-dicarbonyls on Rh atoms supported on NiAl(110).^26^ Furthermore, the approximately two-fold higher intensity of the mixed-isotope 2068 cm^−1^ band relative to the pure ^13^CO dicarbonyl feature is fully consistent with statistical expectations: in an equimolar ^12^CO/^13^CO mixture, the probability of forming Rh_2fold_(^13^CO)(^12^CO) is twice that of forming Rh_2fold_(^13^CO)_2_ or Rh_2fold_(^12^CO)_2_.

*Gem*-dicarbonyls exhibit two characteristic C–O stretching modes, a symmetric and an asymmetric stretch,^18,22,27,69,70^ but we have only discussed the symmetric stretch so far. In powder catalysts, both bands appear as minima in relative reflectivity (*i.e.*, upward absorbance peaks) because the crystallites are randomly oriented with respect to the incident IR light. On a single-crystal surface, the sign and magnitude of Δ*R*/*R*_0_ depend on dipole orientation, light polarisation, and incidence angle, as described by the Fresnel equations (Fig. S1). Perpendicular dipole components give strong negative bands, whereas in-plane dipoles generate much weaker positive features. The symmetric stretch of the Rh_2fold_(CO)_2_*gem*-dicarbonyl has a net dipole-moment change normal to the surface and therefore appears as the negative peak (2037 cm^−1^ for ^13^CO). In contrast, the asymmetric stretch exhibits an in-plane dipole moment change and would appear as a very weak, positive band. In a recent IRAS study of Rh *gem*-dicarbonyls on TiO_2_(110), Eder *et al.*^62^ observed the asymmetric stretch as a weak positive peak ≈61 cm^−1^ below the symmetric stretch peak, in excellent agreement with DFT predictions. Thus, in our spectra, the asymmetric stretch would be extremely weak compared with the symmetric stretch at 2037 cm^−1^ and would fall below the noise level. Moreover, its expected frequency coincides almost exactly with the intense Rh_2fold_CO monocarbonyl band at 1979 cm^−1^, which lies 58 cm^−1^ below the symmetric stretch—very close to the offset reported by Eder *et al.*^62^ and to values known from powder catalysts.^22,69,70^

The 0.4 ML spectrum in Fig. 2 shows that the relative intensities of both the high-frequency band at 2059 cm^−1^ and the intermediate band at 2037 cm^−1^ increase more strongly with Rh coverage than that of the dominant Rh_2fold_CO band (see Table S1). This behaviour is fully consistent with our peak assignment. For the Rh_5fold_ embedded in the surface, it has been shown that its occurrence markedly increases with an increase of the Rh coverage to 0.5 ML.^39^ Likewise, Rh_2_ dimers become more abundant at higher Rh coverages. Initially, deposited Rh atoms diffuse until they are trapped at isolated twofold sites. With increasing coverage, a larger fraction of these sites is already occupied, increasing the probability that an incoming Rh atom encounters and binds to an existing Rh_2fold_ adatom. The resulting dimers are thermodynamically stable, being ≈0.19 eV more stable than isolated Rh_2fold_ adatoms,^46^ which leads to a higher concentration of Rh_2fold_(CO)_2_*gem*-dicarbonyls upon CO exposure under UHV conditions, where *gem*-dicarbonyls are created by dimer decay.^46^ To determine whether *gem*-dicarbonyls also remain a minority species at elevated pressures, we carried out complementary near-ambient-pressure STM measurements, which are presented in the next section.

## 5. Near-ambient-pressure STM: sequential CO adsorption at elevated pressure

**Fig. 5:**
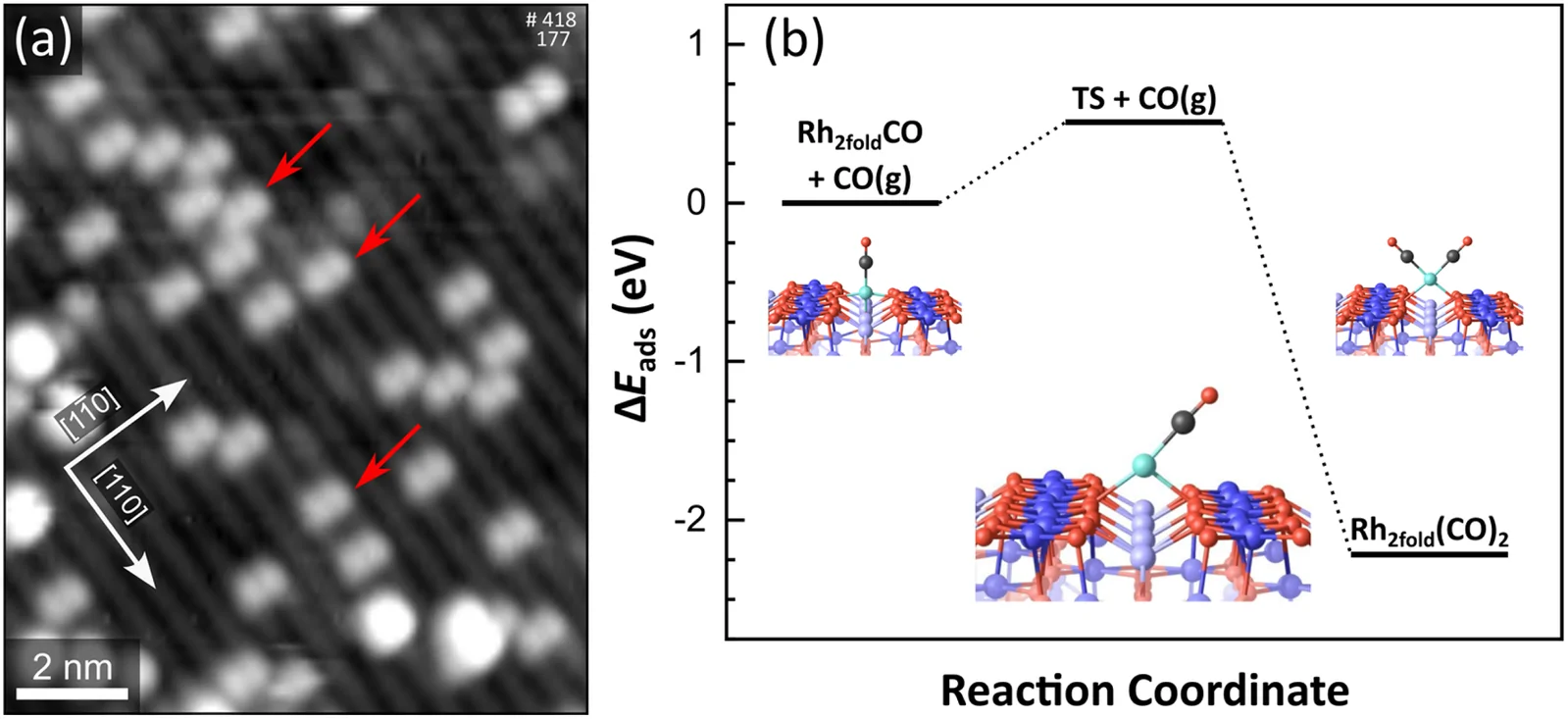
Exposure of Rh/Fe_3_O_4_(001) to 2 mbar CO. (a) NAP-STM image (*V*_sample_ = 2.2 V; *I* = 0.02 nA) of 0.2 ML Rh/Fe_3_O_4_(001) under 2 mbar CO. Two-lobe features are observed as the dominant species and are assigned to *gem*-dicarbonyls formed by adsorption of a second CO molecule on Rh_2fold_CO species (red arrows in Fig. 1a). (b) Adsorption of a second CO molecule onto the monocarbonyl configuration proceeds *via* a metastable transition state (TS), occupied ≈10^−8^ or 10^−9^ of the time at room temperature, as deduced from DFT calculations in our previous study.^46^ Energies are given relative to the monocarbonyl ground state.

As described in Section 3, in UHV experiments, dicarbonyls form *via* dissociation of Rh_2_ dimers.^46^ To test whether this restriction persists at higher pressures, we examined CO adsorption on Rh/Fe_3_O_4_(001) using NAP-STM. After standard UHV preparation and a brief low-dose CO exposure (≈3 L), STM imaging confirmed that the surface contained primarily monocarbonyl Rh_2fold_CO species, as in all previous UHV studies. When a much higher CO dose was applied, 2 mbar for 3 min at room temperature, the surface changed dramatically. STM images recorded at 2 mbar CO [Fig. 5a] showed that essentially all Rh_2fold_CO species adopt a double-lobed geometry identical to the Rh_2fold_(CO)_2_*gem*-dicarbonyls observed in UHV only after CO-induced dissociation of Rh_2_ dimers [red arrows in Fig. 5a]. Under these near-ambient conditions, however, the *gem*-dicarbonyls appear in the absence of any monocarbonyl neighbours (which would result from the Rh-dimer mediated mechanism of dicarbonyl formation), demonstrating that the dicarbonyls now form directly from isolated Rh_2fold_CO monocarbonyls. These *gem*-dicarbonyls were also observed after dosing was stopped and the chamber pumped to UHV conditions, in agreement with the stability of dicarbonyl species inferred from the adsorption energy (Fig. S2). A blank experiment on clean Fe_3_O_4_(001) showed no adsorption, confirming that the species observed originate from Rh.

Based on TPD results,^71^ we estimate that the residence time of a CO molecule on the Fe_3_O_4_(001) surface at RT is in the nanosecond regime. This implies that CO can diffuse in a weakly bound precursor state before being captured at a Rh site. This is consistent with the rapid saturation of the monocarbonyl peak observed in CO exposure-dependent IRAS spectra (66% saturation is achieved already at 0.063 L exposure, Fig. S3). By fitting the uptake curve, we determined the effective capture area per Rh_2fold_ site to be approximately 4 nm^2^, which means that a large fraction of the CO arriving at the surface is efficiently captured at Rh sites in the initial regime. This is consistent with our STM observations, allowing for a moderate tip-shadowing effect (Fig. 1 and Movie S1). Since sequential CO adsorption to form Rh_2fold_(CO)_2_ species was not observed experimentally by STM under UHV conditions, even though CO encounters pre-existing Rh_2fold_CO roughly every 10 seconds at 10^−8^ mbar, we conclude that the capture of the second CO molecule on an isolated monocarbonyl is a rare event. This suggests that the second CO is not generally stabilised upon encounter with Rh_2fold_CO unless the local Rh-support geometry is transiently in a capture-ready configuration. Assuming the same 4 nm^2^ capture zone, each Rh_2fold_CO experiences roughly 2 × 10^7^ CO encounters per second at 2 mbar. Thus, the immediate observation of Rh_2fold_(CO)_2_ at 2 mbar CO in NAP-STM suggests that the increase in the encounter rate renders the requisite rare events accessible on the experimental timescale, leading to the formation of thermodynamically stable Rh_2fold_(CO)_2_ species.

## 6. Discussion

The results presented here demonstrate that IRAS experiments on single crystals under well-defined conditions can provide quantitatively reliable, site-resolved vibrational fingerprints for oxide-supported single-atom catalysts. Using Rh/Fe_3_O_4_(001) as an exemplary model system, we resolve three distinct Rh–CO species, namely monocarbonyls and *gem*-dicarbonyls of twofold-coordinated Rh adatoms, as well as monocarbonyls formed at fivefold-coordinated Rh atoms embedded in the surface. We could assign the vibrational frequencies *ν*_CO_ unambiguously to these species by combining isotope substitution, annealing experiments, STM/nc-AFM imaging, XPS, TPD, and DFT-based calculations. The coordination-dependent shifts in *ν*_CO_ provide a direct handle on the local environment of Rh and can be used as a reference when analysing the more complex CO-IR spectra of supported Rh powder catalysts.^18,24,25,28,69,70^

The present study also exemplifies how single-crystal IRAS relates to CO-IR spectra on powders. On powder catalysts, randomly oriented crystallites and averaging over all incidence angles mean that both symmetric and asymmetric *gem*-dicarbonyl stretches typically appear as bands of increased absorption.^18,24,25,28,69,70^ On the single-crystal Rh/Fe_3_O_4_(001) model system, by contrast, the fixed surface orientation and chosen incidence angle range make the IRAS intensity strongly dependent on dipole direction: components normal to the surface dominate, while in-plane components contribute only weak, oppositely signed features. This explains why we only observe the symmetric stretch of the *gem*-dicarbonyl and do not observe the asymmetric mode, even though both are prominent in powder spectra. While the spectra therefore differ in the intensities of the IR bands, the fingerprint positions of the IR-active species remain unaffected, making UHV-based IRAS studies valuable for identifying, assigning and benchmarking surface species.

The comparison with theory highlights both the strengths and limitations of current DFT approaches. DFT+U calculations correctly predict the relative ordering of *ν*_CO_ for the Rh_2fold_CO and Rh_2fold_(CO)_2_ species at twofold-coordinated Rh adatom sites, and the calculated adsorption energies rule out *gem*-dicarbonyl formation at Rh_5fold_ sites. On the other hand, the calculated difference between the symmetric stretch of Rh_2fold_(CO)_2_ and the CO stretch frequency at Rh_5fold_ strongly depends on details of the computational approach (whether a Hubbard *U* is used for the Rh), so assigning these bands purely on the basis of the calculated frequencies would be ambiguous. Since the CO frequency depends on the charge of the Rh,^66^ we attribute this sensitivity to the different charge states of Rh at these sites: At twofold sites, Rh_2fold_ is singly charged,^46^ while the calculated Bader charge of the Rh_5fold_ is 1.27*e* (1.23*e* with *U*_eff,Rh_ = 3.61 eV), indicating a Rh^2+^ or Rh^3+^ state. Usually, the Rh^3+^ oxidation state is more common, but less likely to form stable carbonyls than Rh^2+^.^66^ In our calculations, the Hubbard *U* mainly shifts the *ν*_CO_ on the Rh_5fold_, while the other configurations are much less affected. Employing *U* is common for similar systems with 3d transition elements,^47^ but it is often neglected for the 4d and 5d metals since several works indicate that *U* values for 4d elements are lower and therefore of less importance.^72,73^ Other work, however, indicates similar *U* values for Fe and Rh.^74^ Given that, in the present system, using *U*_eff,Rh_ = 3.61 eV significantly improves the agreement between calculated and experimental *ν*_CO_ at Rh_5fold_, it may be worthwhile to re-examine the role of Hubbard corrections for 4d (and possibly 5d) elements more generally, even though a single case study is clearly insufficient to justify a universal prescription. It should be noted, however, that the CO adsorption energies calculated with *U*_eff,Rh_ = 3.61 eV (Table 1) are inconsistent with our TPD results.^42,47^

Since the configuration corresponding to each frequency can be unambiguously determined by our experiments, the experimental spectra provide a valuable benchmark for refining computational treatment of metal–CO bonding on oxides. More broadly, the combination of well-defined model surfaces, high-quality IRAS, and complementary microscopy across the UHV–near-ambient range offers a general strategy for understanding the CO-IR signatures of isolated metal sites in real catalysts. This allows identification of which structural motifs and formation pathways are actually relevant under working conditions.

A central mechanistic question concerns the formation of *gem*-dicarbonyls. Under UHV, *gem*-dicarbonyl Rh_2fold_(CO)_2_ species arise almost exclusively from CO-induced dissociation of Rh_2_ dimers, even though the formation of a dicarbonyl from an isolated monocarbonyl is thermodynamically favourable. This behaviour reflects a strong kinetic constraint: sequential adsorption of a second CO molecule onto a Rh_2fold_CO appears to require that CO arrives when the Rh–CO complex is transiently in a capture-ready configuration, a circumstance that is rarely realised at the low encounter rates present during UHV dosing. In our previous work,^46^ we identified one plausible example of such a capture-ready configuration in the form of a metastable geometry approximately 0.5 eV above the Rh_2fold_CO ground state [Fig. 5b]. An energy difference of 0.5 eV corresponds to a configurational probability on the order of 10^−8^–10^−9^ at 300 K, which is consistent with the absence of sequential Rh_2fold_(CO)_2_ formation from isolated Rh_2fold_CO under UHV conditions, while at millibar CO pressures the dramatically increased encounter rate renders such rare configurations accessible on experimentally relevant timescales. As shown by the NAP-STM experiments in Section 5, this restriction is lifted at 2 mbar CO, where *gem*-dicarbonyls form directly on isolated Rh_2fold_ sites and dominate the surface coverage. While the present data do not uniquely identify the capture-ready configuration and additional pathways may also contribute, the combination of UHV and NAP provides a coherent picture in which UHV acts as a kinetic filter that isolates individual reaction channels, whereas near-ambient measurements reveal which pathways dominate under conditions closer to applied catalysis.

## 7. Conclusions

The results demonstrate how IRAS can serve as a bridging technique between idealised model systems and applied systems under realistic conditions. Using Rh/Fe_3_O_4_(001) as a model system in UHV, we resolved and assigned the key Rh carbonyl species present on the surface: monocarbonyls and *gem*-dicarbonyls at twofold-coordinated Rh sites, as well as monocarbonyls formed by fivefold-coordinated Rh embedded in the surface. Under UHV conditions, *gem*-dicarbonyls are a minority species since they form almost exclusively *via* CO-induced dissociation of Rh_2_ dimers, because the sequential adsorption of two CO molecules on an isolated Rh_2fold_ is kinetically blocked despite being thermodynamically favoured. Near-ambient-pressure STM shows that at millibar CO pressures this kinetic limitation is lifted and *gem*-dicarbonyls form directly on isolated Rh_2fold_ sites, reconciling UHV model studies with the *gem*-dicarbonyl signatures commonly observed under catalytic conditions. The experimental CO stretch frequencies are valuable as fingerprints for oxide-supported single-atom catalysts. Comparison with DFT+U highlights that theory does not yet match experimental frequencies quantitatively, underlining the value of the IRAS benchmarks established here for interpreting CO-IR spectra and refining theoretical descriptions of metal–CO bonding on oxide-supported single-atom catalysts.

## Author contributions

NEHB contributed to the investigation, performed formal analysis, data curation, visualisation, and prepared the original draft. CW assisted with data analysis and investigation. PS carried out the theoretical and methodological work, contributed to formal analysis and visualisation. DR supported methodology and contributed to experimental investigation. AL, FL, MO, and LP performed experimental investigation. ZJ contributed to investigation and formal analysis. FK performed data analysis and visualisation. ME contributed to methodology. MS, UD, MM, and CF contributed to the methodology and validation. PM contributed to investigation and methodology. JP contributed to conceptualisation, supervision, validation, and funding acquisition. GSP contributed to conceptualisation, supervision, validation, funding acquisition, and writing of the original draft. All authors participated in reviewing & editing of the manuscript.

## Conflicts of interest

There are no conflicts to declare.

## Supplementary Material

FD-267-D5FD00158G-s001

FD-267-D5FD00158G-s002

## Data Availability

The data supporting this article have been included as part of the supplementary information (SI). Supplementary information is available. See DOI: https://doi.org/10.1039/d5fd00158g.
