# Supplementary material for: CO on a Rh/Fe3O4 single-atom catalyst: high-resolution infrared spectroscopy and near-ambient-pressure scanning tunnelling microscopy
Source: Faraday Discuss. 2026 May 22. Online ahead of print. doi: 10.1039/d5fd00158g (PMC13196844; doi:10.1039/d5fd00158g)
Supplement: FD-OLF-D5FD00158G-s001 [file FD-OLF-D5FD00158G-s001.zip › 2026-01-29_si_revised.docx]

CO on a Rh/Fe₃O₄ single-atom catalyst: high-resolution infrared spectroscopy and near-ambient-pressure scanning tunnelling microscopy

Nail El Hocine Barama,^a^ Chunlei Wang,^a^ Panukorn Sombut,^a^ David Rath,^a^ Adam Lagin,^a^ Martin Ormoš,^b^ Lena Puntscher,^a^ Faith J. Lewis,^a^ Zdeněk Jakub,^a,c^ Florian Kraushofer,^a^ Moritz Eder,^a^ Matthias Meier,^d^ Michael Schmid,^a^ Ulrike Diebold,^a^ Cesare Franchini,^d,e^ Peter Matvija,^b^ Jiří Pavelec*^a^ and Gareth S. Parkinson^a^

Received 00th January 20xx, Accepted 00th January 20xx
DOI: 10.1039/x0xx00000x


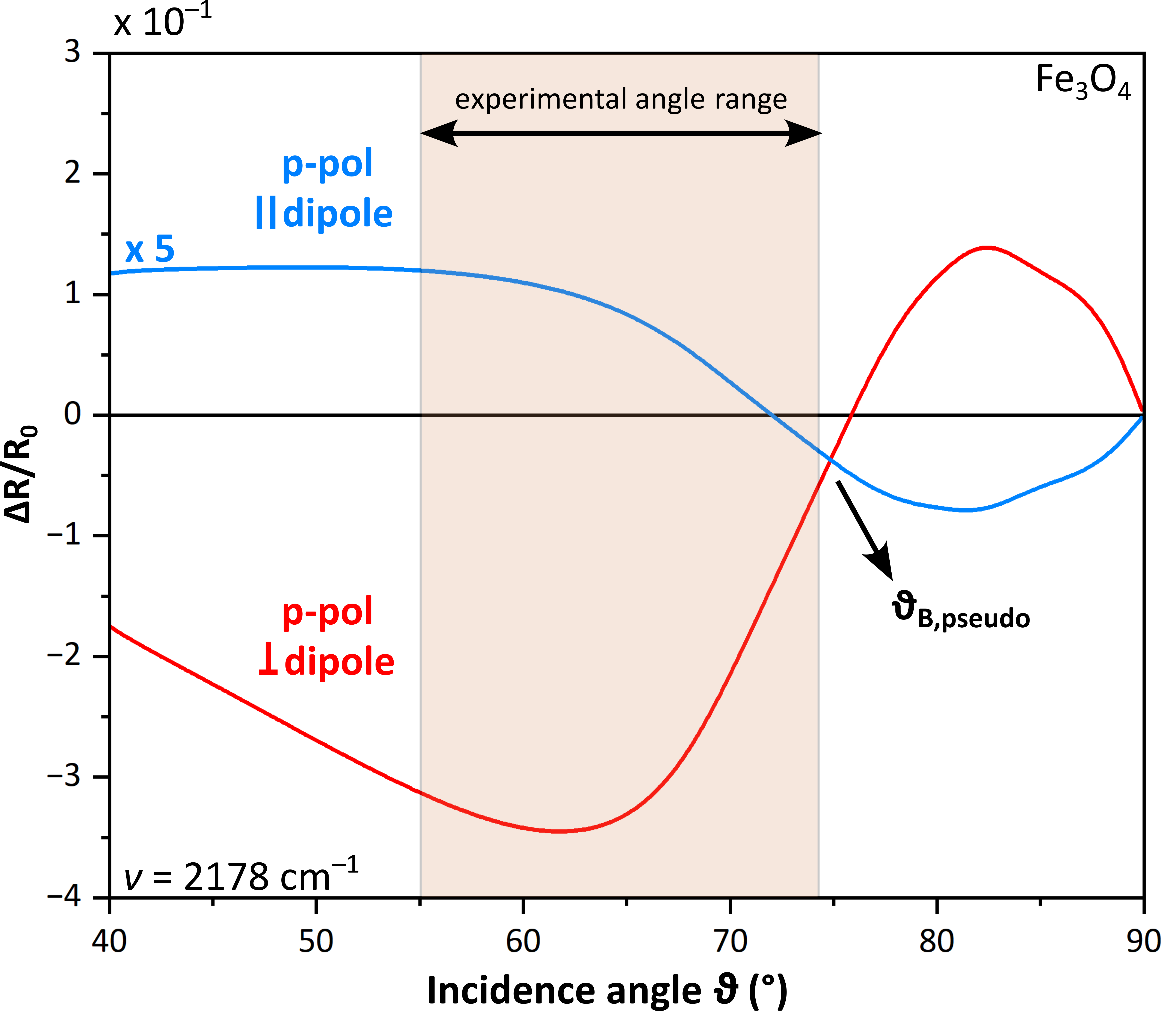
Supporting Information

**Figure S1. Normalised reflectivity difference Δ*R*/*R*_0_ for an adsorbate on Fe_3_O_4_ at a representative frequency of *ν* = 2178 cm^–1^, including system-specific corrections.** Δ*R*/*R*_0_ was calculated using a static electronic polarisability *α_e_* = 0, and a vibrational polarisability *α_v_* = 3.4 × 10^−26^ cm^3^. The calculation includes corrections for an incidence-angle spread of *κ* = ±2°, a system-specific depolarisation factor *Γ* = 0.21, and the contribution from vicinity illumination.^1,2^ The red curve shows the response for p-polarised light interacting with a dipole moment perpendicular to the surface, while the blue curve corresponds to p-polarised light with a dipole moment parallel to the surface and lying in the plane of incidence (scaled by a factor of 5 for clarity). The beige region indicates the incidence-angle range used in our experiments (55°–74°), which lies below the pseudo-Brewster angle where the characteristic band inversion occurs. Within this range, perpendicular dipoles give rise to strong negative peaks, whereas parallel dipoles yield much weaker positive features—approximately fifteen times smaller in magnitude. The signal in p-polarisation vanishes for a dipole moment normal to the plane of incidence. Since the azimuthal orientation of alternating (001) planes of Fe_3_O_4_(001) changes by 90°, only half of the molecules with a given azimuthal orientation with respect to the Fe rows contribute to the signal. This leads to an effective factor of 30 in peak height between out-of-plane and in-plane dipole moments.

**Table S1.** Relative increase in IRAS peak intensities between 0.2 ML and 0.4 ML Rh/Fe₃O₄(001)

| **Peak Position** | **Peak height (0.2 ML Rh)** | **Peak height (0.4 ML Rh)** | **Relative increase** |
| --- | --- | --- | --- |
| 1979 | −5.4 x 10^−4^ | −7.9 x 10^−4^ | ≈1.5 |
| 2037 | −8.8 x 10^−5^ | −3.8 x 10^−4^ | ≈4.3 |
| 2059 | −5.9 x 10^−5^ | −3.0 x 10^−4^ | ≈5.1 |

The table summarises the change in IRAS peak intensities between Rh coverages of 0.2 ML (black spectrum in Fig.2) and 0.4 ML (blue spectrum in Fig. 2). The band at 1979 cm^–1^, associated with CO bound to twofold coordinated Rh_2fold_ sites, increases by a factor of ≈1.5. In comparison, the gem-dicarbonyl symmetric stretch at 2037 cm^–1^ increases by a factor of ≈4.3, and the band at 2059 cm^–1^ attributed to CO adsorption on Rh_5fold_ sites increases by a factor of ≈5.1. The substantially larger relative increases of the higher-frequency bands further support their assignment to gem-dicarbonyl species and CO bound to Rh_5fold_ sites.


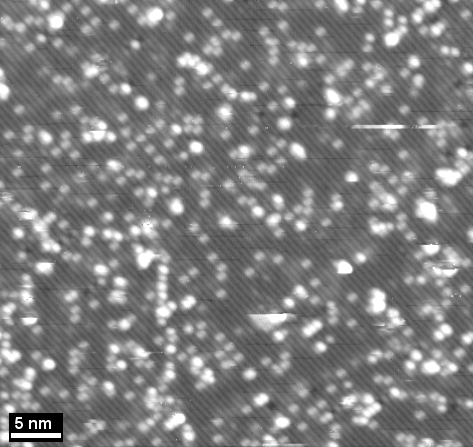


**Figure S2. STM measurement of CO adsorption on Rh/Fe_3_O_4_(001).** This STM image was acquired under UHV conditions following the NAP-STM measurement of Rh/Fe_3_O_4_(001) in 2 mbar CO shown in Figure 5a. The dicarbonyl species formed during CO exposure remain stable even under UHV, consistent with the thermodynamic stability predicted by DFT (adsorption energy: −2.21 eV per CO). The few clusters or blurry features visible in the image may arise from minor contamination deposited during the prolonged CO exposure at 2 mbar.

**
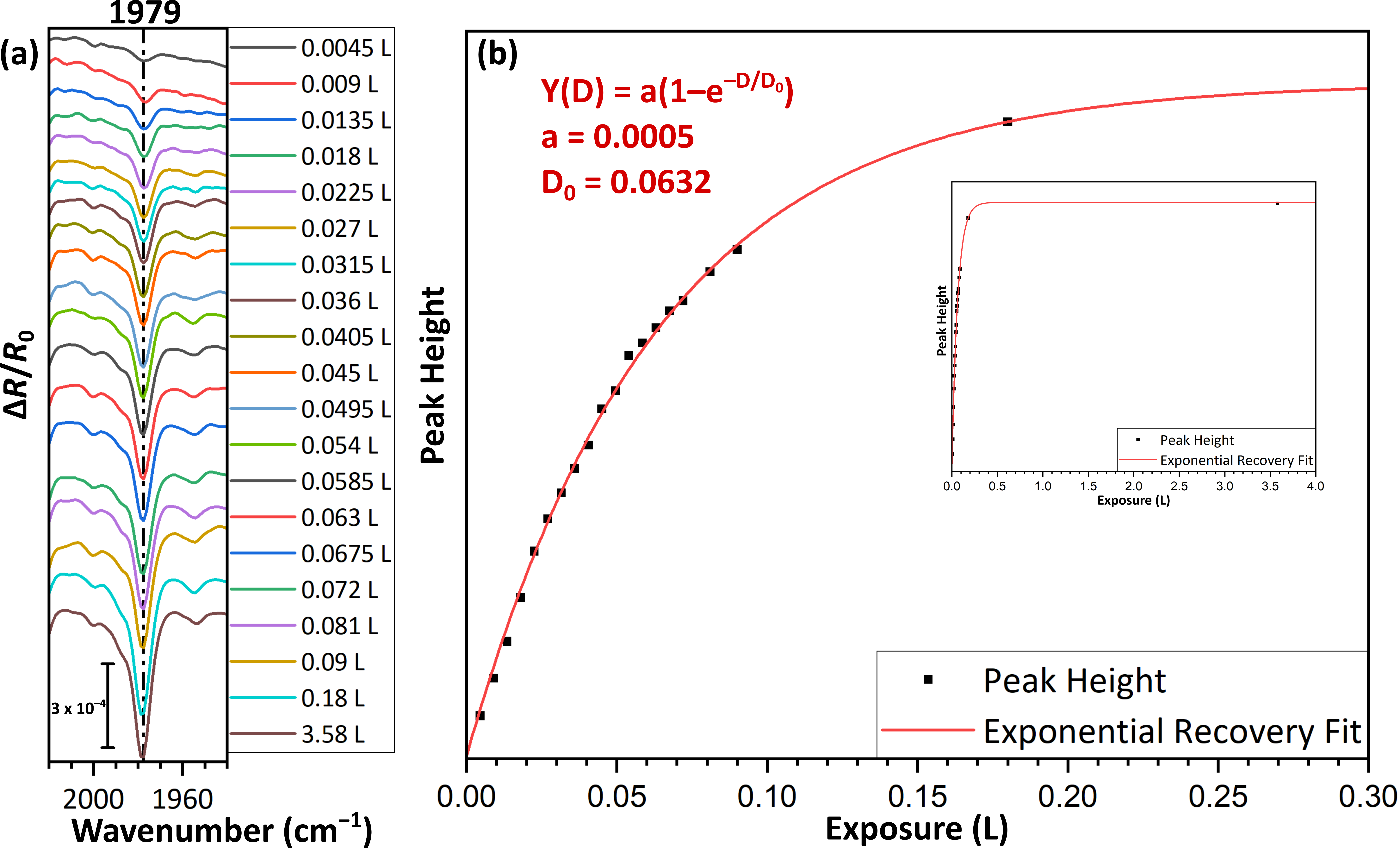
**

**Figure S3. CO exposure-dependence of the Rh_2fold_CO IRAS band.** (a) p-polarised IRAS spectra of a Rh-decorated Fe_3_O_4_(001) surface with a Rh coverage of 0.2 monolayer (ML; 1 ML = 1.42 × 10^14^ Rh atoms per cm^2^) upon exposure to increasing doses of ^13^CO. The reference spectrum R_0_ was recorded after Rh deposition and prior to the first CO dose of 0.0045 Langmuir (L; 1 L = 1.33 x 10^−6^ mbar.s) and was used as a background for all spectra. Already after the first exposure, a weak Rh_2fold_CO band is visible. With successive 0.0045 L increments, the Rh_2fold_CO peak increases progressively in intensity and approaches saturation at a total exposure of ≈0.18 L. (b) Rh_2fold_CO IRAS peak height as a function of CO exposure (black squares), together with an exponential recovery fit (red curve) of the form $y(D)=a[1-exp(-D/D_{0})]$from which a value of $D_{0}$ = 0.0632 is extracted and used to estimate the effective capture area of Rh_2fold_ sites.

The adsorption of CO on Rh/Fe_3_O_4_(001) was quantified by monitoring the IRAS band intensity of Rh_2fold_CO as a function of CO exposure $D$ (in Pa.s). The approach to saturation is well described by a single-exponential function, $y(D)=a[1-exp(-D/D_{0})]$, where $D_{0}$is the characteristic exposure at which $1-1/e$ (63%) of the total maximum peak height is reached.

This behaviour is consistent with first-order adsorption within a Langmuir-type model, for which the fractional coverage of Rh_2fold_CO sites is given by

$\theta(D)=1-exp[-S_{0}ID/{(Pn}_{a})]$,

where $\theta$ is the fraction of Rh_2fold_ sites occupied by CO, $S_{0}$ is the initial sticking coefficient, $I$ is the CO impingement rate (number of CO molecules per unit area and time), and $n_{a}$ is the number of Rh atoms per area. We express the initial sticking coefficient with an effective capture area $A_{c}$ per Rh atom as $S_{0}= A_{c}n_{a}$. This effective capture area can be much larger than the geometric size of a Rh atom if the CO molecule can diffuse as a precursor before being captured by a Rh atom. Comparison with the exponential fit yields $A_{c}=P/(D_{0}I)$. This comparison is valid because the Rh_2fold_CO species are well isolated under the present conditions, and the IRAS peak intensity is proportional to the CO coverage on Rh_2fold_ sites.

From kinetic gas theory, the CO impingement rate is given by

$I=P/\sqrt{2\pi mk_{B}T}$,

where $m$is the mass of a CO molecule, $k_{B}$is the Boltzmann constant, and $T=300$K. This gives $A_{c}=\sqrt{2\pi mk_{B}T}/D_{0}.$ Using the experimentally determined value $D_{0}=0.063 L=8.38 \times{10}^{-6}$Pa.s, we obtain an effective capture area of $A_{c}=4.1$ nm^2^.

This analysis indicates that CO adsorption on Rh_2fold_ sites is highly efficient, with each Rh atom effectively capturing CO molecules incident within its associated capture area.

When compared to the geometric area per Rh atom estimated from the nominal Rh density at 0.2 ML coverage ($n_{\mathrm{Rh}}=0.2\text{ ML}\times1.42\times{10}^{14}\text{ }$cm^−2^), which corresponds to an average area of 3.52 nm^2^ per Rh atom, the extracted capture area is slightly larger. This would correspond to a sticking coefficient of 1.16, which is unphysical. We attribute this discrepancy mainly to the uncertainty of the Rh coverage deposited (10–20%) and of the CO dose (≈10%). In addition, not all Rh atoms are isolated adatoms in twofold sites, but only the Rh_2fold_ contributes to the IRAS peak at 1979 cm^−1^. In any case, the Langmuir model is inaccurate for precursor-mediated adsorption with a capture area close to the area per adsorption site. Nevertheless, since the fit of the IRAS intensities vs. dose assuming Langmuirian adsorption is reasonable, the capture area derived from this model should be useful as a rough estimate. If the effective capture area were much larger, i.e. if the precursor molecules could visit many Rh sites before leaving the surface, the IRAS peak height as a function of dose would not follow the exponential recovery function observed in experiment but increase almost linearly up to saturation.

In principle, one could also try to estimate the CO sticking probability from the STM movies. This would reduce the uncertainty in the density of adsorption sites $n_{a}$, since one could count these sites in the STM images. Nevertheless, this method of estimating the sticking probability is very unreliable, since shadowing by the tip strongly reduces the local impingement rate in the area scanned.

References

1 D. Rath, Infrared Reflection Absorption Spectroscopy with Angle Selection, Technische Universität Wien, 2024.

2 D. Rath, V. Mikerásek, C. Wang, M. Eder, M. Schmid, U. Diebold, G. S. Parkinson and J. Pavelec, *Rev. Sci. Instrum.*, 2024, **95**, 065106.
