# Supplementary material for: CO on a Rh/Fe3O4 single-atom catalyst: high-resolution infrared spectroscopy and near-ambient-pressure scanning tunnelling microscopy
Source: Faraday Discuss. 2026 May 22. Online ahead of print. doi: 10.1039/d5fd00158g (PMC13196844; doi:10.1039/d5fd00158g)
Supplement: FD-OLF-D5FD00158G-s002 [file FD-OLF-D5FD00158G-s002.pdf]

Supplementary Information (SI) for Faraday Discussions.
This journal is © The Royal Society of Chemistry 2026

Faraday Discussions

PAPER

CO on a Rh/Fe_3O_4 single-atom catalyst: high-resolution infrared spectroscopy and near-ambient-pressure scanning tunnelling microscopy

Nail El Hocine Barama,[a] Chunlei Wang,[a] Panukorn Sombut,[a] David Rath,[a] Adam Lagin,[a] Martin Ormoš,[b] Lena Puntscher,[a] Faith J. Lewis,[a] Zdeněk Jakub,[a,c] Florian Kraushofer,[a] Moritz Eder,[a] Matthias Meier,[d] Michael Schmid,[a] Ulrike Diebold,[a] Cesare Franchini,[d,e] Peter Matvija,[b] Jiří Pavelec*[a] and Gareth S. Parkinson[a]

Supplementary Information

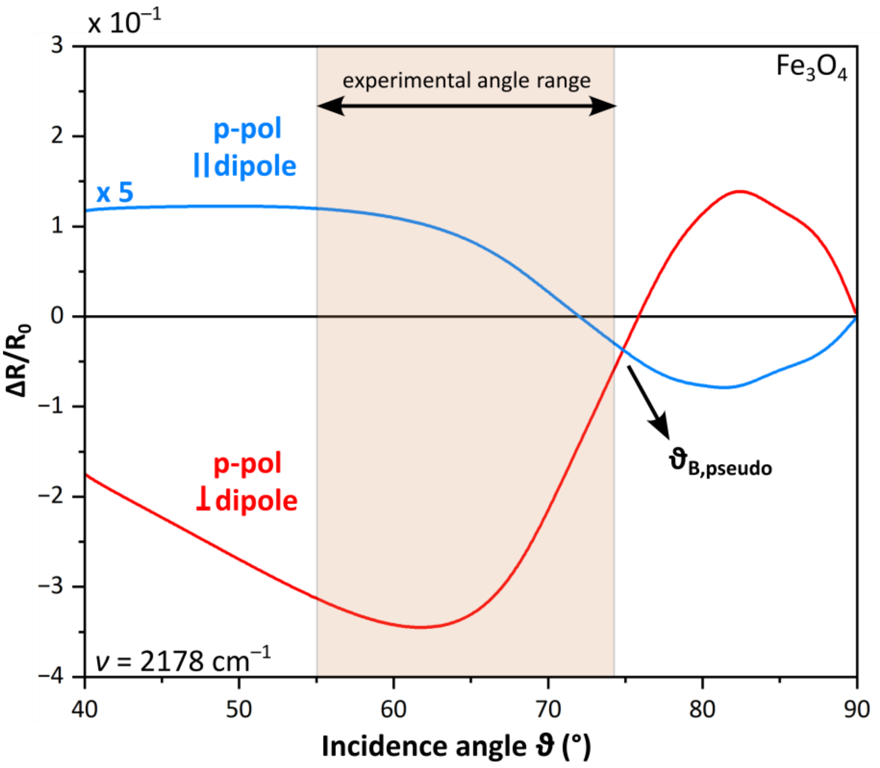

Figure S1. Normalised reflectivity difference $\Delta R/R_0$ for an adsorbate on Fe_3O_4 at a representative frequency of ν = 2178 cm^{-1}, including system-specific corrections. $\Delta R/R_0$ was calculated using a static electronic polarisability α_e = 0, and a vibrational polarisability $\alpha_v = 3.4 \times 10^{-26}$ cm^3. The calculation includes corrections for an incidence-angle spread of $\kappa = \pm 2°$, a system-specific depolarisation factor $\Gamma = 0.21$, and the contribution from vicinity illumination (outside the adsorbate-covered region of ≈3.5 mm diameter).[1,2] The red curve shows the response for p-polarised light interacting with a dipole moment perpendicular to the surface, while the blue curve corresponds to p-polarised light with a dipole moment parallel to the surface and lying in the plane of incidence (scaled by a factor of 5 for clarity). The beige region indicates the incidence-angle range used in our experiments (55°–74°), which lies below the pseudo-Brewster angle where the characteristic band inversion occurs. Within this range, perpendicular dipoles give rise to strong negative peaks, whereas parallel dipoles yield much weaker positive features—approximately fifteen

times smaller in magnitude. The signal in p-polarisation vanishes for a dipole moment normal to the plane of incidence. Since the azimuthal orientation of alternating (001) planes of $Fe_3O_4(001)$ changes by 90°, only half of the molecules with a given azimuthal orientation with respect to the Fe rows contribute to the signal. This leads to an effective factor of 30 in peak height between out-of-plane and in-plane dipole moments.

Table S1. Relative increase in IRAS peak intensities between 0.2 ML and 0.4 ML Rh/Fe_3O_4(001)

Peak Position	Peak height (0.2 ML Rh)	Peak height (0.4 ML Rh)	Relative increase
1979	-5.4×10^{-4}	-7.9×10^{-4}	≈1.5
2037	-8.8×10^{-5}	-3.8×10^{-4}	≈4.3
2059	-5.9×10^{-5}	-3.0×10^{-4}	≈5.1

The table summarises the change in IRAS peak intensities between Rh coverages of 0.2 ML (black spectrum in Fig.2) and 0.4 ML (blue spectrum in Fig. 2). The band at 1979 cm^{-1}, associated with CO bound to twofold coordinated Rh_{2fold} sites, increases by a factor of ≈1.5. In comparison, the gem-dicarbonyl symmetric stretch at 2037 cm^{-1} increases by a factor of ≈4.3, and the band at 2059 cm^{-1} attributed to CO adsorption on Rh_{5fold} sites increases by a factor of ≈5.1. The substantially larger relative increases of the higher-frequency bands further support their assignment to gem-dicarbonyl species and CO bound to Rh_{5fold} sites.

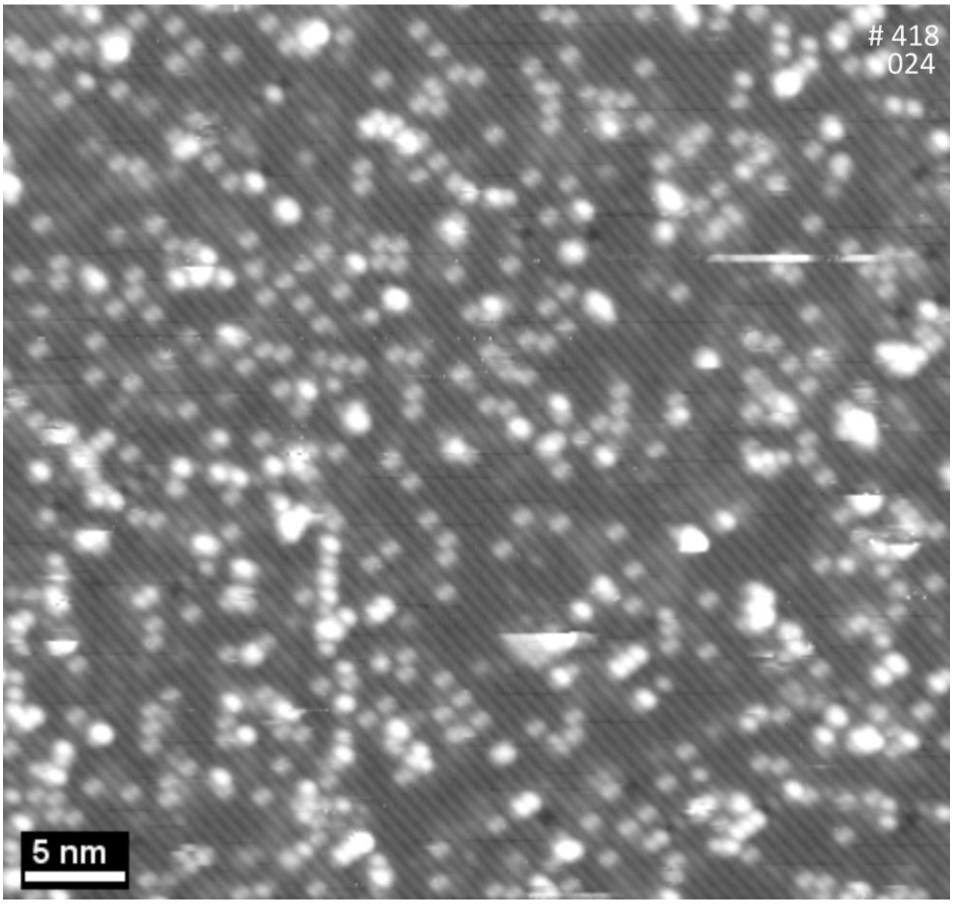

Figure S2. STM measurement of CO adsorption on Rh/Fe_3O_4(001). This STM image (V_{sample} = 2.2 V; I = 0.02 nA) was acquired under UHV conditions following the NAP-STM measurement of Rh/Fe_3O_4(001) in 2 mbar CO shown in Figure 5a. The dicarbonyl species formed during CO exposure remain stable even under UHV, consistent with the thermodynamic stability predicted by DFT (adsorption energy: −2.21 eV per CO). The few clusters or blurry features visible in the image may arise from minor contamination deposited during the prolonged CO exposure at 2 mbar.

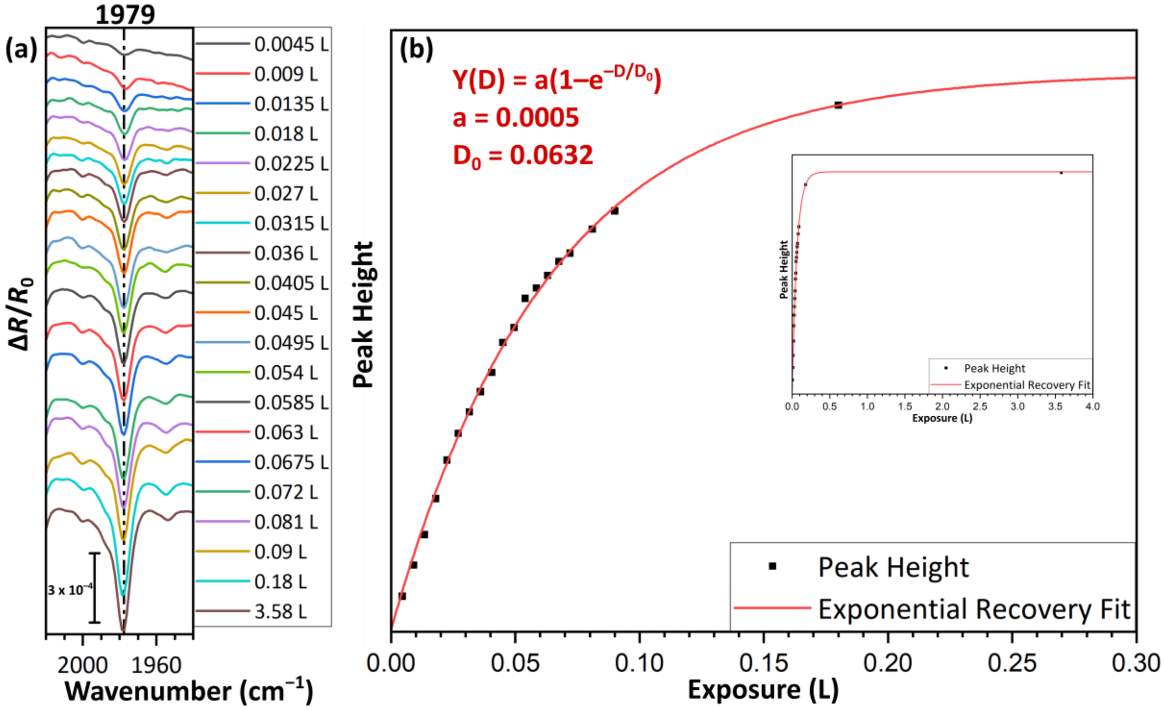

Figure S3. CO exposure-dependence of the $Rh_{2fold}CO$ IRAS band. (a) p-polarised IRAS spectra of a Rh-decorated $Fe_3O_4(001)$ surface with a Rh coverage of 0.2 monolayer (ML; 1 ML = 1.42 × 10^{14} Rh atoms per cm^2) upon exposure to increasing doses of ^{13}CO. The reference spectrum R_0 was recorded after Rh deposition and prior to the first CO dose of 0.0045 Langmuir (L; 1 L = 1.33 × 10^{-6} mbar.s) and was used as a background for all spectra. Already after the first exposure, a weak $Rh_{2fold}CO$ band is visible. With successive 0.0045 L increments, the $Rh_{2fold}CO$ peak increases progressively in intensity and approaches saturation at a total exposure of ≈0.18 L. (b) $Rh_{2fold}CO$ IRAS peak height as a function of CO exposure (black squares), together with an exponential recovery fit (red curve) of the form $y(D) = a[1 - exp(-D/D_0)]$ from which a value of D_0 = 0.0632 L = 8.4 10^{-6} Pa·s is extracted and used to estimate the effective capture area of Rh_{2fold} sites.

The adsorption of CO on $Rh/Fe_3O_4(001)$ was quantified by monitoring the IRAS band intensity of $Rh_{2fold}CO$ as a function of CO exposure D (in Pa·s). The approach to saturation is well described by a single-exponential function, $y(D) = a[1 - exp(-D/D_0)]$, where D_0 is the characteristic exposure at which $1 - 1/e$ (63%) of the total maximum peak height is reached.
This behaviour is consistent with first-order adsorption within a Langmuir-type model, for which the fractional coverage of $Rh_{2fold}CO$ sites is given by

$$\theta(D) = 1 - \exp\left[-S_0 I D/(P n_a)\right],$$

where θ is the fraction of Rh_{2fold} sites occupied by CO, S_0 is the initial sticking coefficient, I is the CO impingement rate (number of CO molecules per unit area and time), P is the pressure, and n_a is the number of Rh atoms per area. We express the initial sticking coefficient with an effective capture area A_c per Rh atom as $S_0 = A_c n_a$. This effective capture area can be much larger than the geometric size of a Rh atom if the CO molecule can diffuse as a precursor before being captured by a Rh atom. Comparison with the exponential fit yields $A_c = P/(D_0 I)$. This comparison is valid because the $Rh_{2fold}CO$ species are well isolated under the present conditions, and the IRAS peak intensity is proportional to the CO coverage on Rh_{2fold} sites.
From kinetic gas theory, the CO impingement rate is given by

$$I = P/\sqrt{2\pi m k_B T},$$

where m is the mass of a CO molecule, k_B is the Boltzmann constant, and $T = 300$ K. This gives $A_c = \sqrt{2\pi m k_B T}/D_0$. Using the experimentally determined value $D_0 = 0.063\ L = 8.38 \times 10^{-6}$ Pa.s, we obtain an effective capture area of $A_c = 4.1$ nm^2.
This analysis indicates that CO adsorption on Rh_{2fold} sites is highly efficient, with each Rh atom effectively capturing CO molecules incident within its associated capture area.

When compared to the geometric area per Rh atom estimated from the nominal Rh density at 0.2 ML coverage ($n_{Rh} = 0.2$ ML $\times 1.42 \times 10^{14}$ cm^{-2}), which corresponds to an average area of 3.52 nm^2 per Rh atom, the extracted capture area is slightly larger. This would correspond to a sticking coefficient of 1.16, which is unphysical. We attribute this discrepancy mainly to the uncertainty of the Rh coverage deposited (10–20%) and of the CO dose (≈10%). In addition, not all Rh atoms are isolated adatoms in twofold sites, but only the Rh_{2fold} contributes to the IRAS peak at 1979 cm^{-1}. In any case, the Langmuir model is inaccurate for

precursor-mediated adsorption with a capture area close to the area per adsorption site. Nevertheless, since the fit of the IRAS intensities vs. dose assuming Langmuirian adsorption is reasonable, the capture area derived from this model should be useful as a rough estimate. If the effective capture area were much larger, i.e. if the precursor molecules could visit many Rh sites before leaving the surface, the IRAS peak height as a function of dose would not follow the exponential recovery function observed in experiment but increase almost linearly up to saturation.

In principle, one could also try to estimate the CO sticking probability from the STM movies. This would reduce the uncertainty in the density of adsorption sites n_a, since one could count these sites in the STM images. Nevertheless, this method of estimating the sticking probability is very unreliable, since shadowing by the tip strongly reduces the local impingement rate in the area scanned.

Notes and references

a. Institute of Applied Physics, TU Wien, 1040 Vienna, Austria
b. Department of Surface and Plasma Science, Charles University in Prague, V Holesovickach 2, 180 00 Praha, Czech Republic
c. Central European Institute of Technology (CEITEC), Brno University of Technology, Brno, 612 00, Czechia
d. Faculty of Physics and Center for Computational Materials Science, University of Vienna, Vienna AT 1090, Austria
e. Dipartimento di Fisica e Astronomia, Università di Bologna, Bologna IT 40126, Italy
* jiri.pavelec@tuwien.ac.at

1 D. Rath, Infrared Reflection Absorption Spectroscopy with Angle Selection, Technische Universität Wien, 2024.
2 D. Rath, V. Mikerásek, C. Wang, M. Eder, M. Schmid, U. Diebold, G. S. Parkinson and J. Pavelec, *Rev. Sci. Instrum.*, 2024, **95**, 065106.
